# Genetic analysis of drought and heat tolerance combined with *Striga* hermonthica resistance in tropical maize (*Zea mays)*

**DOI:** 10.1371/journal.pone.0340288

**Published:** 2026-02-09

**Authors:** Melkamu Elmyhun, Ermias Abate, Alemu Abate, Adefris Teklewold, Silvestro Meseka, Abebe Menkir

**Affiliations:** 1 Department of plant science, College of Agriculture and Environmental science, Bahir Dar University, Bahir Dar, Ethiopia; 2 Adet Agricultural Research Center, Amhar Regional Agricultural Research Institute (ARARI), Adet, Ethiopia; 3 Sasakawa Africa Associations (SAA), Addis Ababa, Ethiopia; 4 International Maize and Wheat Improvement Center (CIMMYT), Addis Ababa, Ethiopia; 5 International Institute of tropical Agriculture (IITA), Nigeria; KGUT: Graduate University of Advanced Technology, IRAN, ISLAMIC REPUBLIC OF

## Abstract

The occurrence of combined biotic and abiotic stresses with more damaging effect is worsening in maize production fields in SSA due to climate change. The development of multiple stress tolerant maize hybrids is thus critical to assure food security. This study was conducted to (i) examine the mode of inheritance of combined tolerance to drought and heat stress (CDHS) with resistance to Striga in tropical maize and (ii) assess the feasibility of selecting hybrids with combined tolerance to drought and heat stress and resistance to Striga infection. Single cross hybrids formed from Striga resistant lines with contrasting resistance reactions to tassel blasting were then evaluated under combined drought and heat stress as well as under Striga infested (STIN) and non-infested (STNO) conditions. The observed high GCA/SCA ratio and narrow sense heritability estimates indicated that additive gene action had a major effect on the inheritance of most traits under all testing conditions. Seven parental lines had positive GCA effects for grain yield under CDHS and STIN conditions when they were used as both male and female parents. We also found single crosses with positive specific combining ability (SCA) for grain yield. Grain yield under STIN had positive and significant genotypic and phenotypic correlations with yield recorded under CDHS and STNO conditions. It thus appears that grain yield may be regulated by common alleles across the three growing conditions. Selected parental lines are potential parents for developing source populations of new inbred lines and superior hybrids with combined CDHS tolerance and Striga resistance. Promising single crosses could be used as female parents to develop multiple stress tolerant 3-way cross hybrids.

## Introduction

Maize is a staple crop contributing significantly to food and nutrition security in Sub Sahara Africa (SSA) [1–3]. Maize occupies 40% to 50% of the total cereal production area in SSA, with more than 80% of the grain being used as food to supply about 30% of the calorie intake to millions of people in the continent [3]. The crop is produced in diverse environments and consumed by people in varying traditional foods with different socioeconomic status [4]. Despite its multitude uses and adaptation to diverse agro ecological zones in SSA, yields of maize in farmers’ field are still low [5], creating significant challenges to food security. Several factors including biotic and abiotic stresses, inadequate use of inputs and improved crop management practices contribute to lower maize grain yields in farmers’ fields [5,6]. Drought, heat stress and *Striga hermonthica* infection are the major limiting factors reducing maize grain yield in SSA. *Striga hermonthica* is a parasite that attaches to host roots and removes essential nutrients resulting in stunted growth and poor yield [7]. *Striga hermonthica* can cause an average yield loss of 68% in maize [8] that can reach complete crop loss under severe infestation. Drought and heat stress negatively affects morphological, physiological, biochemical and molecular aspects of maize leading to poor growth and low yields. Drought and heat stress affects different stages of maize, including germination, vegetative development, flowering, and grain filling and also reduces leaf photosynthesis and enhances leaf senescence rate [9,10]. The reproductive stage of maize is highly susceptible to drought and heat stress causing up to 66% yield reduction [11].

The co-occurrence of stresses inflicts more severe yield losses compared to the effect of individual stresses [11–13]. Concurrent occurrence of Drought, heat stress and *Striga* infection has presented complex challenge to maize production in SSA. Substantial investment in breeding for host plant resistance for many years led to the development of *Striga hermonthica* resistant maize [14–16] germplasmontrast, drought and heat stress tolerant maize variety development was started after initiation of heat stress tolerant studies in 2019 [17]. The development of maize varieties with combined tolerance to the two stresses is therefore important to boost productivity in areas affected by the concurrent presence of these stresses in smallholder farmers’ fields [12,18–20]. Extensive testing of new inbred lines in hybrid combinations is essential to assess their combining abilities for selecting suitable parents for developing high yielding and stress resilient maize hybrids [21–24]. Several studies have been conducted to determine the mode of inheritance of grain yield under individual stress, including drought and heat stress tolerance and *Striga* resistance in maize, showing inconsistent results. Elmyhun et al. [25], reported the predominance of additive gene actions regulating yield under drought and heat stress while Akula et al. (2016) reported the preponderance of non-additive gene actions controlling grain yield and most yield related traits. Amegbor et al.[26] and Sangaré et al. [14]  reported the importance of additive gene action controlling yield under *striga* infestation, whereas Okoth, [27] and Annor et al., [28]  found non-additive gene actions playing a prominent role in conditioning this trait. However, limited studies have been conducted to determine the mode of inheritance of combined tolerance to drought and heat stress and *Striga* infection. This study was therefore conducted to (i) examine the genetic basis of tolerance to combined drought and heat stress and *Striga* infection in tropical maize and (ii) assess the potential to select hybrids with combined tolerance to the two stresses.

## Materials and methods

### Description of genetic materials

As a CGIAR center, IITA introduced germplasm with no restrictions from diverse sources. These sources were then used by breeders to develop *Striga* resistant lines through continual screening using artificial *Striga* infestation. Many S1 to S3 lines, derived from bi-parental crosses of elite drought-tolerant and *Striga*-resistant white maize inbred lines, were subjected to a sequential selection scheme. In this process, lines at each inbreeding generation were first screened under artificial *Striga* infestation followed by evaluation of the selected resistant lines under managed drought stress until the S4 lines were developed. Additionally, S1 to S3 lines, derived from backcrosses involving temperate lines (4402/4401, LH59-N1, and LH82-N1) as donors of desirable traits and elite drought-tolerant and *Striga*-resistant lines as recipients, were also subjected to the same sequential selection scheme. As a result of this selection, 56 lines with desirable agronomic traits, minimal leaf firing, and tolerance to tassel blasting were identified, along with 21 lines that were susceptible to tassel blasting, for further evaluation in hybrid combinations. These lines were subsequently tested at Kadawa station in 2017 to confirm their responses to combined heat and drought stress. The results of this evaluation formed the basis for selecting 12 tassel blast-tolerant and 12 tassel blast-susceptible lines for the present study (S1 Table). These 24 IITA inbred lines, owner by the center, were split into 6 groups, each containing four inbred lines. The four inbred lines from one group were used as mothers and crossed with the four inbred lines from another group acting as fathers utilizing the NCII. Each inbred line served as the female parent in one group and as the male parent in another group. The trial included 96 single-cross hybrids (comprising 6 sets of 16 hybrids each), two standard *Striga* tolerant and susceptible lines (9022−13 and 8338−1) and the commercial hybrids (OBA SUPER 7 and OBA SUPER 9), which were not bred for tolerance to drought and heat stress were used as checks, evaluated under combined drought and heat stress (https://doi.org/10.25502/cw1h-kw05/d) conditions with artificial *Striga* infested (STIN) and *Striga* non-infested ((https://doi.org/10.25502/kpt0-e749/d and) conditions.

### Evaluation of Maize Performance under Combined Drought and Heat Stress (DHS)

The tolerance of 100 maize hybrids to simultaneous DHS and drought stress was assessed at Kadawa (11^0^390 N, 8^0^270 E, and elevation 500 m) in Kano State, Nigeria, experiencing temperatures ranging from 33 to 45°Celsius from February to June in 2020 and 2021. Kadawa's soil is classified as Regosols, with a texture ranging from sandy to clay loam, pH of 5.9, organic carbon content of 4.3 g kg^−1^, and residual nitrogen level of 0.24 g kg^−1^ [29]. These conditions allowed for hybrid evaluation under deficit irrigation by carefully controlling water supplies through furrow irrigation. The flowering and seed development phases occurred in April during a period of minimal rainfall and low humidity of 15% to 46%, leading to drought conditions and high temperatures affecting the hybrids. Maize hybrids were planted in mid-February in both 2020 and 2021 with tasseling and silking taking place in mid-April, which coincides with the hottest period in Kadawa. The hybrids were set up in 25 × 4 alpha lattice layouts with two replications and were initially cultivated in well-watered settings before being subjected to heat and drought stress simultaneously. A furrow irrigation method was employed to deliver enough water to the crops every four days in the initial 45-day period following planting. Irrigation was stopped in mid-April when the average daytime temperature ranged from 36 to 45°Celsius and nighttime temperature ranged from 27 to 30° Celsius, aligning with the flowering period of the hybrids. After three weeks, hybrids were irrigated once a week to help them fill their grains until they reached physiological maturity.

### Evaluation of Maize Performance under artificial *Striga*- infested (STIN) and non-infested (STNO) conditions

The 100 hybrids were also evaluated under artificial *S. hermonthica* infestation and non-infested conditions at Kubwa and Mokwa in Nigeria during the main rainy seasons in 2020 and 2021 using a 25 × 4 alpha lattice design. Kubwa and Mokwa are located 340 km apart and represent different climatic conditions. Kubwa is located near Abuja (9°14’N and 7°35’E, 445 m elevation), with a ferric luvisol Plinthustalf soil containing 81% sand, 12% silt, and 7% clay, receives about 1389 mm of rainfall, and has average monthly minimum temperatures of 19–24° C and maximum temperatures of 26–34° C during evaluation of the hybrids for two years. At Kubwa, the growing season starts in May and ends in October. Mokwa is located in Niger state (9°29’N and 5°05’E, 153 m elevation), with a Tropeptic Haplustox soil that is fine and kaolinitic in nature, receives 1150 mm of rainfall, and has average monthly minimum temperatures of 20–23° C and maximum temperatures of 29–34° C during evaluation of the hybrids for two years. The growing season at Mokwa starts at the beginning of July and ends in October.

Each hybrid was planted in adjacent infested and non-infested strips facing opposite to each other and separated by 1.5 m alley. Within each strip, the same hybrid was planted in an infested row and a non-infested row to determine precise estimates of yield losses due to *S. hermonthica* damage. Each row was 5 m long with a spacing of 0.75 m between rows and 0.25 m spacing between plants within a row. The non-infested rows were treated with ethylene two weeks before planting to stimulate the germination of *S. hermonthica* seeds present in the soil to keep the land free of Striga seeds. Every year, *S. hermonthica* seeds were collected from farmers’ sorghum fields around Abuja and Mokwa and used for infestation, which was carried out by injecting 8.5 g of sand-mixed *S. hermonthica* seed innoculum into the holes of about 6 cm deep and 10 cm wide. About 3,000 germinable *S. hermonthica* seeds were placed in each hill. Two maize seeds were placed into each hole infested with sand-mixed *S. hermonthica* seeds and covered with soil. One plant was manually removed from each hill two weeks after planting to attain a population density of 53,333 plants ha^-1^. As *S. hermonthica* infection is high under low nitrogen, a compound fertilizer was applied at the rate of 30 kg ha^-1^ N, 60 kg ha^-1^ P, and 60 kg ha^-1^ K at planting, with additional 30 kg ha^-1^ N applied four weeks later. Weeds other than *S. hermonthica* were removed by hand throughout the cropping season.

### Trait measurements

Important traits were recorded under DHS, STIN and STNO conditions. Days to 50% anthesis and silking were recorded when half of the plants within a plot showed extruded anther and produced silks, respectively. Anthesis-silking interval was computed as days to 50% silking minus days to 50% anthesis. Plant and ear heights were recorded as the average heights of five representative plants in each plot measured from the base of the plant to the node bearing the tassel and the upper ear, respectively. Ears per plant were taken as the ratio of total number of ears harvested divided by the total number of plants in each plot. Plants showing tassel blasting and leaf firing symptoms were counted for DHS plots. Striga damage rating (SDR) was visually recorded using a 1–9 scale where 1 = no observable damage, normal maize growth and high level of resistance, and 9 = total death of the maize plant [30], in each infested plot at 8 and 10 weeks after planting. Also, the emerged *S. hermonthica* plants were counted in each infested plot at 8 and 10 weeks after planting. Plant aspect was recorded based on the assessment of general physical appearance of plants in each plot using a scale of 1–5, where 1 = outstanding general phenotypic appearance and 5 = totally unattractive phenotypic appearance. Ear aspect was rated based on a scale of 1–5, where 1 = clean, uniform, large and entirely filled ears without disease/insect damage symptoms, and 5 poorly filled ears with disease and insect damage. Husk cover was rated based on a scale of 1–5, where 1 = husks tightly arranged and extended above the ear tip and 5 = exposed ear tips. Grain yield in kg ha^–1^ was determined from field weight and percent moisture recorded in each plot using 80% shelling percentage and adjusted to 15% moisture content.

### Statistical data analysis

A mixed model was used to perform a combined analysis of variance (ANOVA) for traits measured under each testing environment (DHS, STIN, and STNO). Each location × year combination was considered as a separate environment. The 96 single-crosses and four checks were treated as fixed effects, whereas environments, replicates within environments, and blocks within replicates were considered as random effects. Line × tester analysis was performed using the cross means generated from the individual ANOVA, excluding checks. Analysis of Genetic Designs with R (AGD-R) was employed to estimate general combining ability (GCA) and specific combining ability (SCA) effects for all recorded traits under each management condition. The proportion of the sum of squares attributable to GCA and SCA effects was calculated to assess the relative importance of additive and dominance gene actions. Genotypic and phenotypic correlations among testing environments for grain yield (GY) and between GY and other traits were computed using META-R [31]. Additionally, stress tolerance indices were estimated according to the formulae summarized in S2 Table.

## Results

### Combined analyses for traits recorded under stressful and non-stressful conditions

Highly significant (p < 0.001) differences were detected among environments, hybrids, and parental lines for grain yield (GY), days to anthesis (DA), days to silking (DS), plant height (PH), and ear height (EH) across Striga-infested (Table 1), Striga non-infested (Table 2), and combined drought and heat stress (DHS) (Table 3) conditions. The environmental effect was the largest contributor to variation in GY, with mean squares of 7.45 × 10⁷, 5.91 × 10⁷, and 3.57 × 10⁸ under Striga-infested, non-infested, and DHS conditions, respectively. Hybrid effects were also significant (MS = 4.07 × 10⁶, 5.35 × 10⁶, and 3.88 × 10⁶), indicating substantial genetic variation among testcrosses. Female and male parental effects were significant for most traits (p < 0.001), while female × male interactions were smaller and often non-significant. The environment × hybrid interaction was significant for GY across all conditions (MS = 1.36 × 10⁶, 1.74 × 10⁶, and 1.73 × 10⁶), demonstrating variability in hybrid performance across test environments.

**Table 1:** Mean squares for maize traits under Striga-infested (STIN) conditions.

Source of Variation	Df	GY	DA	DS	PH	EH
ENV	3	74526218***	2764***	2675***	10795***	0.59ns
REP(ENV)	4	16849148***	17***	21***	4062***	2124***
BLK (ENV × REP)	192	1479712***	7***	8***	259***	132*
SET	5	6928152***	11***	13***	2014***	565***
ENV × SET	15	1282430***	3	4*	108	88
HYBRID	95	4071753***	22***	23***	627***	156**
FEMALE(SET)	18	3702470***	25***	26***	618***	180*
MALE(SET)	18	2804581***	18***	17***	333***	163*
FEMALE × MALE(SET)	54	1566202***	4***	5***	112	108
ENV × HYBRID(Set)	285	1362200***	4***	7***	254***	162**
ENV × FEMALE(SET)	54	811802	2	3	112	197*
ENV × MALE(SET)	54	1208184***	3*	4***	124	345***
ENV × FEMALE × MALE(SET)	162	591394	2	3	110	108
ERROR	188	578141	2	2	94	94

**Table 2:** Mean squares for maize traits under Striga-non-infested (STNO) conditions.

Source of Variation	Df	GY	DA	DS	PH	EH
ENV	3	59051466***	2998***	2891***	9763***	58491***
REP(ENV)	4	9260301***	111***	115***	1977***	968***
BLK (ENV × REP)	192	1766043***	8***	8***	548***	211***
SET	5	7325228***	15***	14***	5250***	1929***
ENV × SET	15	827652	3	3	662*	97
HYBRID	95	5346615***	23***	24***	1453***	574***
FEMALE(SET)	18	4186352***	21***	23***	910***	444***
MALE(SET)	18	5640607***	18***	19***	769***	490***
FEMALE × MALE(SET)	54	812208*	4***	4***	331	78
ENV × HYBRID	285	1736888***	6***	6***	551***	184***
ENV × FEMALE(SET)	54	974588***	3	4*	403	97
ENV × MALE(SET)	54	760455	3	3*	358	148
ENV × FEMALE × MALE (SET)	162	644535	3	3	343	91
ERROR	188	546268	3	2	313	106

**Table 3:** Mean squares for maize traits under combined drought and heat stress (DHS) conditions.

Source of Variation	Df	GY	DA	DS	PH	EH
ENV	1	356879627***	512***	530***	63697***	24019***
REP(ENV)	2	6099191***	24***	33***	115	107
BLOCK (REP × ENV)	96	6862290***	10***	13***	322***	173***
SET	5	5956558***	14***	14***	1197***	961***
ENV × SET	5	5062839***	6	7	111	126
HYBRID	95	3880039***	12***	72	378***	378***
FEMALE (Set)	18	6046756.3***	25***	30***	438***	275***
MALE (Set)	18	5036613***	17***	18***	239***	218***
FEMALE × MALE (Set)	54	1506140***	2	5*	102*	46ns
ENV × HYBRID(Set)	95	1728089***	3	66	178***	171***
ENV × FEMALE (Set)	18	1378870*	3	6*	77	30
ENV × MALE(Set)	18	2965651***	4	4	56	75
ENV × FEMALE × MALE (Set)	54	1052136ns	3	4	81	53
ERROR	102	746465	3	3	66	60

Where GY = grain yield, DA = days to 50% anthesis, DS = days to 50% silking, PH = plant height, and EH = ear height, *, **, and *** significant at p < 0.05, P < 0.01 and p < 0.001.

### Combining ability, variance component and heritability estimates

Variance for females (δ2F) was larger than the variances for male (δ2 M) and female by male interactions (δ2MF) for all agronomic traits, expect yield under STIN and STNO conditions (Table 2). Genetic variances (δ2G) for these traits were larger than environmental variances (δ2E) under all testing conditions. The variances for general combining ability (δ2GCA) and additive (δ22*A*) effects of GY, ANT, SIL, PH, EH, PASP, EASP, EPP and SLB were larger than those of the specific combining ability (δ2SCA) and dominance (δ2D) effect under STIN, STNO and DHS conditions. The high narrow sense heritability (ℎ2) estimates and Baker ratio for GY, ANT, SIL, PH, EH, PASP, EASP, EPP and SLB recorded under all growing conditions implied the preponderance of additive gen action regulating the traits (Tables 4 and 5).

**Table 4:** Genetic parameters for yield and its components in environments with and without Striga harmonica infestation.

Genetic component	Measured traits											
	STINF						STNON					
	GY	DA	DS	EASP	SC2	SDR2	GY	DA	DS	PH	PASP	EASP
δ^2^M	51555	0.72	0.67	0.014	14.22	0.087	238882	0.81	0.84	41.01	0.01	0.021
δ^2^F	132522	1.06	1.01	0.017	13.46	0.116	207312	0.92	0.94	65.83	0.02	0.0.21
δ^2^MF	192597	0.38	0.46	0.012	1.78	0.037	545370	0.37	0.35	1.45	0.04	0.011
δ^2^G	361839	2.00	2.04	0.041	28.75	0.24	476144	1.98	2.00	112.3	0.06	0.049
δ^2^E	109285	0.30	0.33	0.016	15.13	0.27	105421	0.31	0.38	52.7	0.06	0.020
δ^2^GCA	723678	4.01	4.09	0.081	57.51	0.485	952281	3.96	4.01	224.8	0.12	0.099
δ^2^SCA	770386	1.50	1.85	0.047	7.11	0.15	218148	1.49	1.41	5.78	0.14	0.042
δ^2^*A*	1447355	8.02	8.18	0.162	115.0	0.97	190458	7.91	8.02	449.5	0.24	0.198
δ^2^*D*	770386	1.50	1.85	0.047	7.11	0.15	218148	1.49	1.41	5.78	0.14	0.042
δ^2^GCA/ δ ^2^SCA	0.94	2.67	2.21	1.72	8.09	3.23	4.37	2.65	2.84	38.88	0.86	2.36
Baker ratio	0.65	0.84	0.81	0.78	0.94	0.86	0.90	0.84	0.85	0.99	0.63	0.83
H^2^	0.95	0.97	0.97	0.93	0.88	0.80	0.95	0.97	0.96	0.90	0.87	0.92
ℎ^2^	0.62	0.82	0.79	0.72	0.88	0.70	0.85	0.81	0.82	0.90	0.55	0.76

Where PASP = Plant aspect, EASP = Ear aspect, SC2 = striga count 8 weeks after planting and SDR2 = striga damage rating 8 weeks after planting

**Table 5:** Genetic component estimates for measured traits under combined drought heat stress conditions.

Genetic									
Component	GY	ANT	SIL	PH	EH	PASP	EASP	EPP	SLB
δ²M	194252.6	1.11	1.30	23.6	26.8	0.02	0.02	0.007	0.003
δ²F	360572.5	1.86	2.02	44.0	28.8	0.03	0.04	0.005	0.015
δ²MF	187780.9	0.20	0.18	9.6	2.5	0.01	0.03	0.003	0.004
δ²G	711050.4	2.96	3.29	83.6	65.7	0.06	0.08	0.014	0.021
δ²E	518870.0	0.80	0.96	22.3	14.1	0.06	0.07	0.011	0.017
δ²GCA	1422100.9	5.92	6.58	167.2	131.3	0.13	1.65	0.029	0.043
δ²SCA	751123.5	0.79	0.72	38.5	10.0	0.04	0.12	0.013	0.017
δ²A	2844201.7	11.84	13.15	334.4	262.6	0.26	0.33	0.057	0.085
δ²D	751123.5	0.79	0.72	38.5	10.0	0.04	0.12	0.013	0.017
δ²GCA/δ²SCA	1.89	7.49	9.13	12.13	13.13	3.25	13.75	2.23	2.53
Baker ratio	0.79	0.94	0.95	0.90	0.96	0.87	0.96	0.82	0.83
H²	0.87	0.94	0.92	0.94	0.95	0.83	0.87	0.87	0.75
h²	0.69	0.88	0.89	0.85	0.95	0.71	0.64	0.71	0.62

### Combining ability effects of parents and single crosses

Significant differences in general combining ability (GCA) effects for grain yield were observed among parental lines across stress conditions (Table 6). Under *Striga*-infested (STIN) conditions, ten parental lines (P5, P6, P7, P10, P14, P16, P18, P19, P21, and P22) showed positive and significant GCA effects when used as both male and female parents. Under *Striga* non-infested (STNO) conditions, seven lines (P5, P9, P10, P14, P18, P19, and P22) exhibited positive GCA effects in both parental effects, while under combined drought and heat stress (DHS), eight lines (P7, P9, P10, P14, P18, P19, P21, and P22) were identified with positive male and female GCA effects. Notably, P7, P10, P14, P18, P19, P21, and P22 maintained positive GCA effects under both STIN and DHS conditions, indicating consistent performance across stress environments. Among these, P7 and P10 were resistant to tassel blasting, whereas P14, P18, P19, P21, and P22 were susceptible, suggesting that tassel blasting resistance was not directly associated with higher GCA for grain yield.

**Table 6:** GCA effect estimates for grain yield in 96 single crosses involving 24 inbred lines with contrasting resistance to tassel blast under STIN, STNO and DHS.

Line	GYSTIN		GYSTNO		GY DHS	
	Female	Male	Female	Male	Female	Male
1	−24.8	−77.1	−66.8	−105.4	−77.4	68.3
2	15.8	−82.4	43.3	−417.4	−459.0	−155.0
3	67.6	−48.8	−66.3	−359.3	−141.1	−150.6
4	−178.0	−91.1	−171.7	−93.6	131.4	−95.6
5	466.5	117.7	220.7	296.0	288.2	−22.4
6	335.7	40.5	376.0	−59.9	28.5	−90.7
7	118.4	85.3	−7.6	83.7	957.08*	167.6
8	−266.5	−290.0	−11.3	−621.1*	−619.2	−348.7
9	−28.7	87.8	264.8	556.2*	186.0	13.2
10	389.4	96.2	478.8	638.7*	243.9	287.7
11	−643.1	−134.3	−893.4***	−615.5*	−360.7	−112.5
12	−487.2	−58.6	−696.9***	−231.8	−687.0	−295.3
13	−402.5	−7.9	−403.4	−129.8	73.5	−154.8
14	238.2	91.7	412.7	220.4	444.7	429.3
15	75.0	−63.2	−100.8	−54.5	−128.9	−18.4
16	84.0	81.9	2.0	330.6	−400.7	131.2
17	−159.5	−83.4	−399.4	−306.7	−363.2	−212.8
18	182.1	179.1	476.4	346.0	120.1	30.8
19	223.9	186.4	389.7	718.1***	1056.3*	670.7
20	16.9	−27.6	−148.5	−91.7	−94.1	−136.1
21	135.1	219.4	426.3	337.6	344.9	368.9
22	236.0	94.6	461.2	731.6***	448.0	245.5
23	−88.1	−142.3	−112.2	−515.5*	−211.7	−258.6
24	−306.1	−173.8	−473.5	−656.8***	−779.6	−361.9

Out of 96 crosses 45 had positive SCA effect for grain yield under STIN (S3 Table). Forty-eight (50%) crosses recorded positive SCA effect for grain yield under STNO condition. The SCA effect of 45 crosses was positive for grain yield under DHS conditions. Of these crosses, 32 exhibited positive SCA effect for grain yield under STIN and DHS conditions (S3 Table). Similarly, 28 crosses showed positive SCA effect for the same trait across all testing conditions. Most of these crosses had non- significant negative SCA effects for days to 50% anthesis and silking across all growing conditions indicating the possibility of developing early maturing single crosses. A tolerant × susceptible hybrid (SC37) and two susceptible x susceptible hybrids (SC82 and SC95) produced higher yields than heat stress tolerant check (CH97) under STIN and STNO conditions (S4 Table). Relative to the heat susceptible check CH98, 13 hybrids recorded significantly higher grain yield under STIN. In contrast, 42% and 60% of the hybrids showed higher grain yields than CH97 and CH98 under STNO conditions, respectively (S4 Table).

Two tolerant x susceptible hybrids (SC41 and SC43) and two susceptible x susceptible hybrids (SC85 and SC95) were competitive to COH 99 but showed higher grain yield than CH97 under DHS condition. Six hybrids produced higher yields than CH98 and COH100 under DHS (S4 Table). Hybrid SC95 had produced consistently higher grain yields than the heat tolerant (CH97) and heat susceptible (CH98) checks across all testing environments indicating the possibility of developing hybrids with tolerance to combined stresses.

The stress tolerance indices GMP, MP, HMP, STI, YSI and YI identified SC37, SC38, SC42, SC82 and SC95 as tolerant hybrids to Striga (S5 Table) while SC41, SC43, SC85 and SC95 as tolerant to combined heat and drought stress (S6 Table). In contrast, these indices identified SC27 and SC64 as the most susceptible hybrids to *Striga,* and hybrids SC24 and SC36 as the most sensitive to DHS. SC73 was found to be the most susceptible to both STIN and DHS.

### Performance of parents in hybrid combinations

All sets showed broader ranges under DHS than under STIN and STNO conditions (Table 7). Additionally, all sets had the lowest average grain yields under DHS conditions. The relative ranking of mean grain yields of the six sets were consistent under all growing conditions, expect for set 3 and set 6 under DHS conditions. Set 3 and Set 6 had the highest mean grain yields, while set 2 had the lowest mean grain yields under all stress conditions. Sets involving tassel blast resistant lines as both male and female parents produced the lowest grain yields, while sets involving tassel blast susceptible lines as one or both parents had higher mean grain yields. It is interesting to note that set 6, which involved tassel blast susceptible lines as male and female parents, produced the highest mean grain yield under DHS condition. High yielding hybrids under DHS formed from heat stress susceptible lines can be attributed to several factors. Lines may possess genetic traits that confer resilience to drought, such as improved water-use efficiency or deep rooting systems. Additionally, heterosis may be attributed to the observed yield improvement, where the hybrids outperform their parents in stress resilience and overall yield potential. The interaction between environmental stress and trade-off mechanisms where plants prioritize yield production over stress resilience could also play a role in maximizing grain yield under DHS conditions. Moreover, under combined stresses, specific genetic pathways may be up regulated, enhancing the plant’s ability to compensate for heat stress and maintain high yields. These findings underscore the complexity of plant stress responses and suggest that breeding for multiple stress tolerance may yield unexpected yet favourable outcomes, emphasizing the need for further exploration in stress-resilient breeding programs.

**Table 7:** Mean grain yields of the different sets evaluated under *Striga* infested and non-infested conditions and combined drought and heat stress.

		Striga Infested			
		Minimum (kgha-1)	Maximum (kgha-1)	Rank	
1	TBR X TBR	2317.9	3939.3	5	3179.8 ± 94.0
2	TBR X TBR	1747.4	3930.3	6	2949. 2 ± 90.4
3	TBS X TBR	2114.3	5012.6	1	3630. 0 ± 86.7
4	TBR X TBS	1893.9	4481.5	4	3355.8 ± 91.4
5	TBS X TBS	1470.3	4376.6	3	3413.3 ± 96.2
6	TBS X TBS	2490.8	5214.0	2	3464.4 ± 81.6
		Non-infested condition			
1	TBR X TBR	2568.6	4453.1	5	3534.6 ± 91.4
2	TBR X TBR	1770.4	4577.5	6	3279.2 ± 87.9
3	TBS X TBR	2482.8	5031.0	1	4041.3 ± 84.3
4	TBR X TBS	2053.8	4773.3	4	3774.4 ± 88.8
5	TBS X TBS	1893.9	4719.3	3	3802.1 ± 93.5
6	TBS X TBS	2790.0	5398.4	2	3857.8 ± 79.4
		Combined drought heat stress			
1	TBR X TBR	467.5	4068.3	5	1890.7 ± 304.8
2	TBR X TBR	245.1	3816.6	6	1781.6 ± 233.3
3	TBS X TBR	304.8	5705.8	2	2658.8 ± 478.6
4	TBR X TBS	631.5	4025.4	4	2385.9 ± 240.2
5	TBS X TBS	274.3	4474.8	3	2483.0 ± 341.1
6	TBS X TBS	418.6	6715.8	1	2875.3 ± 451.3

TBR = Tassel blast tolerant, TBS = Tassel blast susceptible.

Tassel blast is a potential factor which has high negative impact on maize production. Hybrids derived from tassel tolerant inbred lines could inherent genes for tolerant, the complexity of the trait, genetic interactions, incomplete dominance, epistatic effects, and the potential loss of specific adaptation could cause to increase susceptibility in hybrids.

### Genetic and phenotypic correlations among growing conditions

Grain yield under STIN condition showed significant and strong genotypic and phenotypic correlations with grain yield under DHS and STNO conditions indicating the presence of common genetic mechanisms governing yield under these conditions (Table 8). The genetic and phenotypic relationships between grain yield recorded under DHS and STNO were also strong and positive, implying that gene expressions were consistent across growing conditions (STIN, STNO and DHS).

**Table 8:** Correlations among growing conditions based on grain yield.

Testing environment Combinations	Phenotypic Correlation	Genetic Correlation
Heat Stress vs *Striga* infestation	0.646***	0.825***
Heat stress vs *Striga* non-infested condition	0.684***	0.872***
*Striga* infested vs non-infested condition	0.844***	0.998***

### Genetic and phenotypic correlations of grain yields with other agronomic traits

Under STIN and STNO conditions, grain yield was negatively and significantly correlated with day to anthesis, days to silking, ear aspect, SDR1 and SDR2 and positively and significantly associated with plant height, ear height, ears per plant, SC1 and SC 2 (Table 9). Similarly, yield under DHS showed negative and significant phenotypic and genetic correlations with anthesis and silking days, ear aspect, plant aspect but had positive and significant phenotypic and genetic correlations with plant height and ear height.

**Table 9:** Phenotypic and genetic correlations of grain yield with other traits measured under stressful and favorable growing conditions.

Trait	Phenotypic Correlation			Genetic Correlation		
	GYSTNO	GYSTIN	GYDHS	GYSTNO	GYSTIN	GYDHS
Days to 50% anthesis	−0.647***	−0.545***	−0.814***	−0.708***	−0.589***	−0.999***
Days to 50% silking	−0.641***	−0.585***	−0.791***	−0.698***	−0.602***	−0.999***
Plant height	0.622***	0.716***	0.651***	0.744***	0.858***	0.753***
Ear height	0.576***	−0.820***	0.632***	0.648***	−0.863	0.999***
Ear aspect	−0.799***	−0.826***	−0.929**	−0.920***	−0.907***	−0.999***
Plant aspect	−0.662***	–	−0.753***	−0.849***	–	−0.999***
Husk cover	−0.135 ns	0.266	−0.673***	−0.150 ns	0.760***	–
*Striga* count 8 weeks after planting (SC1)	–	0.231*	–	–	0.452***	–
*Striga* count 10 weeks after planting (SC2)	–	0.253*	–	–	0.463***	–
*Striga* damage 8 weeks after planting (SDR1)	–	−0.432***	–	–	−0.589***	–
*Striga* damage 10 weeks after planting (SDR2)	–	−0.411***	–	–	−0.595***	–

## Discussion

Maize growth and development stages are negatively impacted by biotic and abiotic stresses, with individual effects being studied to select maize genotypes tolerant to each stress [32]. Studies on plant responses to combinations of two or more stresses are limited due to the complicated interactions between multiple stresses. Tesfaye et al. [33] demonstrated that combining drought and heat stress tolerance into tropical maize varieties can boost grain yield under both the current and future climate conditions. Developing maize hybrids combining tolerance to combined DHS and resistance to Striga requires understanding of complex interaction between DHS and Striga infection. Identifying maize varieties with combined stress tolerance is important for areas affected by simultaneous presence of two or more stresses. Our present study has thus evaluated agronomic performance of hybrids formed from lines with contrasting resistance to tassel blast under DHS, STIN and STNO conditions. Significant differences were detected among hybrids under DHS, STIN and STNO conditions, indicating the potential to select hybrids with tolerance to combined stresses without compromising yield under STNO condition [34]. The significant variability observed in hybrids also enabled the selection of multiple traits that confer resistance to various stresses. The high heritability estimates for grain yield and other traits under all testing environments indicates the strong and consistent genetic component of these traits for multiple stress tolerance and optimum environments. Consistent results reported in [34–36]. It also indicated that maize hybrids had the potential for consistent performance across both stressful and favourable growing environments.

The high GCA variance relative to the SCA variance and Baker’s ratio for most traits measured under all testing conditions indicate greater influence of additive gene action on the inheritance of most traits. Similar the findings were observed in other studies [37,38–40]. The GCA effect is therefore important for identification of potential parents to form source populations or derive new stress tolerant maize inbred lines [29,41] as well as develop superior hybrids under combined stresses [39,42]. High GCA variance highlights the potential of inbred lines to contribute favourable alleles for developing hybrids adapted to multiple environments [29,42]. High GCA is beneficial for breeding programs to successfully select and combine parents to create better inbred lines for developing high-yielding and resilient hybrids under both optimal and stressed environments. Furthermore, the predominance of additive genetic effects for yield and other agronomic traits indicates the feasibility of using recurrent selection methods to generate robust parental lines to develop high yielding maize hybrids [17]. High GCA variance implied that hybrid performance can be predicted based on the performance of their parents and result predictable, consistently high performing hybrids without specific combination [17,38,42]. Two tassel blast and *Striga* resistant and five *Striga* and tassel blast susceptible parental lines had positive GCA effects for grain yield across all testing environments, consistent with results of other studies [43–47]. These parental lines generated the highest yielding hybrids with good SCA effects. The presence of similar combining ability effects of these inbred lines under individual and combined stress conditions highlight the feasibility of identifying parents with tolerance to combined DHS and STIN [48–50]. Hybrids yielded 10% less under STIN relative to STNO conditions, implying that the Striga resistant parental lines sustained less yield loss in hybrids under parasite pressure. Eight tassel blast resistant x resistant, eleven tassel blast susceptible x resistant and nine tassel blast susceptible x susceptible single cross hybrids displayed positive SCA effects for grain yield under stressful and non-stressful conditions. Some studies recommend use of at least one resistant parent to develop high yielding hybrids [43–47]. In contrast, other studies found that crossing two susceptible parents could generate high yielding hybrids under stressful and optimum environments [19,51,52] possibly due to the presence of recessive resistance genes in susceptible parents [53]. This paradox may be explained by the presence of recessive resistance or stress-tolerance alleles in the parental lines that are homozygous at different loci. While these inbreeds remain susceptible due to the lack of complementary alleles, their hybrids can combine multiple recessive alleles to express tolerance through genetic complementation [53]. Furthermore, the enhanced performance of such hybrids may be attributed to epistatic interactions among resistance loci, as well as non-additive genetic effects, including dominance and over dominance, which are known contributors to heterosis in maize [54,55]. These findings underscore the importance of cryptic genetic variation and gene interactions in developing stress-resilient hybrids from seemingly susceptible parental lines.

Combined stress tolerance of hybrids was measured by yield superiority of hybrids obtained under each and combined stress condition compared to hybrid checks. A blast resistant x susceptible hybrid (SC37) and two blast susceptible x susceptible hybrids (SC82 and SC95) were the highest yielders compared to the heat resistant and susceptible checks [51] under STIN and STNO environments. The combination of different alleles from the two parents exhibits heterosis which may mask the susceptibility and enhance the resistant traits in hybrids [53,56]. Resistant hybrid from susceptible parents could be due to over dominance gene action, homozygosity of recessive resistance genes inherited from susceptible parents, epistatic interactions, or unique physiological adaptations to stress environments [57,58]. Two blast resistant x susceptible (SC41 and SC43) and two susceptible x blast susceptible hybrids (SC85 and SC95) produced higher grain yields than CH97, CH98, COH100 and were competitive to COH 99 under DHS condition. Hybrids derived from blast resistant x susceptible and blast susceptible x susceptible crosses had yield advantages over the benchmark hybrid in another study [39,52].

In our study, sets containing hybrid is involving tassel blast resistant lines as either male or female parent resulted in the lowest grain yields in our study of contrast, sets hybrids involving tassel blast susceptible lines as one or both parents achieved higher average grain yields. Resistant hybrids could be formed from resistant x susceptible parental line crosses under stress conditions [19,43,56]. In contrast, Musembi et al. [59] reported high yielding hybrids developed by combing two drought susceptible lines under drought and well-watered conditions, suggesting parents carried drought tolerant genes as heterozygous recessive. Schaffasz et al.[51] also found high mid parent heterosis in crosses formed from two cold susceptible sorghum parents. In another study, hybrids formed from crosses of salt tolerant maize inbred lines did not generate salt tolerant hybrids [49]. Salt tolerance in hybrids formed from salt-tolerant maize inbred lines may not be observed due to non-additive gene actions, such as dominance or epistasis, that disrupt the expression of tolerance traits in hybrids, while pleiotropic effects may introduce trade-offs between traits important to salt tolerance [60]. Additionally, hybridization could interfere with critical gene networks responsible for salt tolerance, leading to reduced effectiveness in the hybrid [61]. Since salt tolerance is a polygenic trait, hybrids might fail to inherit enough small-allelic effects originating from both parents to exhibit strong tolerance, and the failure to combine complementary salt-tolerant alleles could further limit its expression [61]. Environmental factors could also mask tolerance traits, underscoring the complex interaction between genetics and environment in the expression of stress responses. It is thus possible to identify susceptible parents that can generate high yielding hybrid under stressful conditions [19].

Strong phenotypic and genetic correlations of yield under STIN with STNO yield under DHS and STNO conditions obtained in our study highlight the presence of common genetic mechanisms controlling reactions of hybrids under optimum and stressful growing conditions. Genes controlling common adaptive responses of genotypes under both stress and non-stress environments were reported in other studies [53,55,61,62]. Pandey et al. ([63]) and Kissoudis et al. ([64]) found genes under combined biotic and abiotic stresses triggering coping mechanisms of plants to multiple abiotic and biotic stresses by creating common genetic mechanisms at physiological and biochemical levels. Strong correlation between stress and optimum growing conditions also indicate that STIN tolerant hybrids could improve their performance across DHS and STNO conditions [32,53,62]. It also implied the presence of broad and more generalized mechanism of stress resilience for developing varieties that can withstand multiple environmental challenges and improve breeding efficiency [53,55,61,62].

Selection of genotypes based on yield performance solely is not always successful as it is a complex trait governed by many genes and affected by genotype by environment interaction especially under stress conditions [65]. Therefore, selection efficiency under stress conditions for grain yield could be improved using tightly linked secondary traits [59,65]. The significant positive genetic and phenotypic correlations of plant and ear heights with grain yield under STNO, DHS and STIN suggest that these characters could be used for yield improvement [66,65,67,68]. Selecting for increased plant and ear heights may use high inputs and synthetization of more assimilates for the production of more grains. This finding align with the current breeding trend toward short-statured maize hybrids with low to mid ear placement, which prioritize input-use efficiency, standability, and stress resilience without compromising yield. Days to 50% tasseling and silking had strong negative genetic and phenotypic associations with grain yield under STIN, STNO and DHS conditions indicating the possibility of developing early maturing hybrids under these growing conditions consistent with the results in other studies [63,65]. The negative phenotypic and genotypic correlations of SDRI and SDR2 with grain yield under STIN indicate their importance in selecting and improving GY under *Striga* infestation [67,68]. Future development of multiple stress tolerant hybrids will rely on genomics, biotechnology, and high-throughput phenotyping. Breeders can hasten the development of multiple stress-tolerant hybrids to enhance resilience of farmers to challenges presented by combined environmental stresses by utilizing cutting-edge tools such as genetic engineering, CRISPR-Cas9, and climate-smart breeding.

## Conclusion

Multiple stress resilient maize hybrids are important for increasing yield in farmers’ fields where combined stresses commonly occur together. Significant variation was observed among single crosses formed from inbred lines with contrasting resistance to tassel blast facilitating identification of single cross hybrids with good levels of tolerance to each and combined stresses. The moderate to high heritability values for grain yield and most other traits under all testing conditions indicated the presence of high potential of traits to transfer from parent to hybrids. The predominance of additive gene action and higher Baker ratio in the inheritance of yield and most other traits under all testing environments indicate the feasibility of selecting lines combining tolerance to DHS and resistance to Striga for inbred recycling and hybrid development. Seven parental lines (P7, P10, P14, P18, P19, P21 and P22) with positive GCA effects for grain yield under all growing conditions when used as both male and female parent can be exploited as donors of combined stress tolerance in tropical maize breeding programs parental lines. These inbred lines can also be used to develop high yielding combined tolerance to DHS and STIN hybrids. Grain yield under STIN had strong, positive genotypic and phenotypic correlations with gain yield under DHS and non-infested (STNO) conditions suggesting that single crosses with tolerance to combined stresses and optimum conditions can be developed. Approaches using genetic tools, including genomics, biotechnology, and high-throughput phenotyping, genetic engineering, CRISPR-Cas9, and climate-smart breeding, will enhance the development of multiple stress tolerant hybrids in the future.

## Supporting information

S1 Table Code for twelve tassel blast tolerant with *Striga* resistant and twelve tassel blast and *Striga* susceptible lines and their cross pattern. (DOCX)

S2 Table Summary of equations and references for stress tolerance indices determination for evaluation of maize single cross hybrids. (DOCX)

S3 Table SCA effect estimates for grain yield and selected yield related traits in 96 single crosses involving 24 lines with varying resistance to tassel blast and *Striga* tested under *Striga* free (STUN), Striga infestation (STIN), combined drought and heat stress conditions (CDHS). (DOCX)

S4 Table Mean performance for grain yield of 100 genotypes (96 hybrids and 4 standard checks) tested under Striga free (STUN), Striga infestation (STIN), combined drought and heat stress conditions (CDHS). (DOCX)

S5 Table Stress tolerant indices under Striga infested environment. (DOCX)

S6 Table Stress tolerant indices under combined heat and drought environment. (DOCX)
