# Correction: Genetic analysis of drought and heat tolerance combined with *Striga* hermonthica resistance in tropical maize (*Zea mays)*

**DOI:** 10.1371/journal.pone.0344712

**Published:** 2026-03-10

**Authors:** The PLOS One Staff

The affiliation for the third author is incorrect. Alemu Abate is not affiliated with #2 but with #1: Department of plant science, College of Agriculture and Environmental science, Bahir Dar University, Bahir Dar, Ethiopia.

The publisher apologizes for the error.
