# Supplementary material for: Genetic analysis of drought and heat tolerance combined with Striga hermonthica resistance in tropical maize (Zea mays)
Source: PLoS One. 2026 Feb 9;21(2):e0340288. doi: 10.1371/journal.pone.0340288 (PMC12885306; doi:10.1371/journal.pone.0340288)
Supplement: S1 Table — (DOCX) [file pone.0340288.s001.docx]

Supplementary table 1 Code for twelve tassel blast tolerant with *Striga* resistant and twelve tassel blast and *Striga* susceptible lines and their cross pattern

|  | | Female |  | Male | Code for their hybrids |
| --- | --- | --- | --- | --- | --- |
| Code | | Heat Stress tolerant S6 lines derived from bi-parental crosses of white DTSTR lines | Code | Heat Stress tolerant lines derived from DTSTR BCs containing temperate germplasm |  |
|  | | SET-1 |  | SET 2 | SC1-SC16 |
| P1 | | TZISTR1198/TZISTR1152-9-1-1-1-B-B | P5 | 1131(LH82-N1)-B/IITATZISTR1108-1-1-1-1-B |  |
| P2 | | TZISTR1198/TZISTR1152-19-1-1-1-B-B | P6 | 1131(LH82-N1)-B/IITATZISTR1108-4-1-1-1-B |  |
| P3 | | TZISTR1198/TZISTR1152-28-1-1-1-B-B | P7 | 1131(LH82-N1)-B/IITATZISTR1108-28-1-1-1-B |  |
| P4 | | TZISTR1199/TZISTR1152-130-1-1-1-B-B | P8 | 1133 LH59-N1-B/IITATZISTR1110-5-1-1-1-B |  |
|  | | Heat Stress tolerant lines derived from DTSTR BCs containing temperate germplasm |  | Heat Stress tolerant S6 lines derived from bi-parental crosses of white DTSTR lines |  |
|  | | SET-3 |  | SET1 | SC17-SC32 |
| P9 | | 1133 LH59-N1-B/IITATZISTR1110-60-1-1-1-B | P1 | TZISTR1198/TZISTR1152-9-1-1-1-B-B |  |
| P10 | | 1133 LH59-N1-B/IITATZISTR1110-62-1-1-1-B | P2 | TZISTR1198/TZISTR1152-19-1-1-1-B-B |  |
| P11 | | 4402 (/),4401-B/IITATZISTR1112-60-2-1-1-B | P3 | TZISTR1198/TZISTR1152-28-1-1-1-B-B |  |
| P12 | | 1133 LH59-N1-B/IITATZISTR1113-27-1-1-1-B | P4 | TZISTR1199/TZISTR1152-130-1-1-1-B-B |  |
|  | | Heat Stress tolerant lines derived from DTSTR BCs containing temperate germplasm |  | Selected lines susceptible to heat stress |  |
|  | | SET-2 |  | SET5 |  |
| P5 | | 1131(LH82-N1)-B/IITATZISTR1108-1-1-1-1-B | P17 | IITATZI1742/TZISTR1106-28-1-1-1-B | SC33-SC48 |
| P6 | | 1131(LH82-N1)-B/IITATZISTR1108-4-1-1-1-B | P18 | 1133 LH59-N1-B/IITATZISTR1110-8-1-1-1 |  |
| P7 | | 1131(LH82-N1)-B/IITATZISTR1108-28-1-1-1-B | P19 | 4402 (/),4401-B/IITATZISTR1112-9-5-1-1 |  |
| P8 | | 1133 LH59-N1-B/IITATZISTR1110-5-1-1-1-B | P20 | 1133 LH59-N1-B/IITATZISTR1113-14-1-1-1 |  |
|  | | Selected lines susceptible to heat stress |  | Heat Stress tolerant lines derived from DTSTR BCs containing temperate germplasm |  |
|  | | SET6 |  | SET3 |  |
| P21 | | 1131(LH82-N1)-B/IITATZISTR1108-19-1-1-1 | P9 | TZISTR1154/TZISTR1111-2-1-1-1-B | SC59-SC64 |
| P22 | | 1131(LH82-N1)-B/IITATZISTR1108-10-1-1-1 | P10 | TZISTR1154/TZISTR1111-34-1-2-1-B |  |
| P23 | | 1131(LH82-N1)-B/IITATZISTR1108-6-4-1-1 | P11 | TZISTR1198/TZISTR1152-140-1-1-1-B |  |
| P24 | | 1131(LH82-N1)-B/IITATZISTR1108-30-2-1-1 | P12 | TZISTR1198/TZISTR1152-162-1-2-1-B |  |
|  | | Selected lines susceptible to heat stress |  | Selected lines susceptible to heat stress |  |
|  | | SET4 |  | SET6 | SC65-SC80 |
| P13 | | 1131(LH82-N1)-B/IITATZISTR1108-19-1-1-1 | P21 | TZISTR1154/TZISTR1111-2-1-1-1-B |  |
| P14 | | 1131(LH82-N1)-B/IITATZISTR1108-10-1-1-1 | P22 | TZISTR1154/TZISTR1111-34-1-2-1-B |  |
| P15 | | 1131(LH82-N1)-B/IITATZISTR1108-6-4-1-1 | P23 | TZISTR1198/TZISTR1152-140-1-1-1-B |  |
| P16 | | 1131(LH82-N1)-B/IITATZISTR1108-30-2-1-1 | P24 | TZISTR1198/TZISTR1152-162-1-2-1-B |  |
|  | | Selected lines susceptible to heat stress |  | Selected lines susceptible to heat stress |  |
|  | | SET5 |  | SET-4 |  |
| P17 | | TZISTR1154/TZISTR1111-2-1-1-1-B | P13 | 1133 LH59-N1-B/IITATZISTR1110-60-1-1-1-B | SC81-SC96 |
| P18 | | TZISTR1154/TZISTR1111-34-1-2-1-B | P14 | 1133 LH59-N1-B/IITATZISTR1110-62-1-1-1-B |  |
| P19 | | TZISTR1198/TZISTR1152-140-1-1-1-B | P15 | 4402 (/),4401-B/IITATZISTR1112-60-2-1-1-B |  |
| P20 | | TZISTR1198/TZISTR1152-162-1-2-1-B | P16 | 1133 LH59-N1-B/IITATZISTR1113-27-1-1-1-B |  |
| Standard checks | | | | | |
| 25 | 9022-13 (Tolerant check) | |  |  | CH97 |
| 26 | 8338-1 (Susceptible check) | |  |  | CH98 |
| 27 | OBA SUPER 7 | |  |  | COH99 |
| 28 | OBA SUPER 9 | |  |  | COH100 |
