# Supplementary material for: Genetic analysis of drought and heat tolerance combined with Striga hermonthica resistance in tropical maize (Zea mays)
Source: PLoS One. 2026 Feb 9;21(2):e0340288. doi: 10.1371/journal.pone.0340288 (PMC12885306; doi:10.1371/journal.pone.0340288)
Supplement: S3 Table — (DOCX) [file pone.0340288.s003.docx]

Supplementary table 3 SCA effect estimates for grain yield and selected yield related traits in 96 single crosses involving 24 lines with varying resistance to tassel blast and *Striga* tested under *Striga* free (STUN), *Striga* infestation (STIN), combined drought and heat stress conditions (CDHS)

| Genotype | GY | | | ANT | | | SIL | | |
| --- | --- | --- | --- | --- | --- | --- | --- | --- | --- |
|  | STUN | STIN | DHS | STUN | STIN | DHS | STUN | STIN | DHS |
| HB1 | 25.0 | 38.5 | -176.0 | 0.47 | 0.41 | 0.1 | 0.37 | 0.54 | 0.83 |
| HB2 | -7.5 | 137.1 | 2.6 | 0.62 | 0.21 | 0.09 | 0.57 | 0.18 | 0.87 |
| HB3 | 22.1 | 259.6 | 84.6 | -0.53 | -0.21 | -0.22 | -0.53 | -0.24 | 0.6 |
| HB4 | 27.9 | 4.6 | 67.1 | -0.53 | -0.31 | 0.01 | -0.35 | -0.37 | 0.96 |
| HB5 | 34.4 | 32.7 | -54.7 | -0.11 | -0.12 | 0.01 | -0.01 | -0.08 | 0.84 |
| HB6 | 66.8 | 179.8 | 71.6 | 0.32 | 0.57 | -0.02 | 0.31 | 0.65 | 0.98 |
| HB7 | -30.1 | -25.8 | -30.6 | 0.09 | 0.21 | 0.09 | 0.06 | 0.09 | 0.91 |
| HB8 | -84.7 | -35.5 | -73.9 | -0.29 | -0.56 | 0.06 | -0.33 | -0.51 | 0.94 |
| HB9 | 37.7 | 162.7 | 428.3 | -0.33 | -0.37 | -0.18 | -0.38 | -0.35 | 0.69 |
| HB10 | -16.8 | 116.9 | -113.8 | -0.30 | -0.52 | 0.04 | -0.29 | -0.73 | 0.81 |
| HB11 | -27.0 | 76.9 | -87.9 | -0.05 | 0.02 | -0.07 | 0.15 | 0.18 | 0.82 |
| HB12 | 25.2 | -37.8 | -64.5 | 0.63 | 0.74 | 0.09 | 0.48 | 0.68 | 0.85 |
| HB13 | -114.7 | -270.0 | -237.9 | 0.21 | 0.35 | 0.04 | 0.28 | 0.27 | 0.96 |
| HB14 | -31.3 | -410.9 | -199.4 | -0.46 | -0.08 | 0.05 | -0.44 | 0.10 | 0.94 |
| HB15 | 17.6 | -212.4 | -39.5 | 0.31 | -0.20 | 0.14 | 0.15 | -0.24 | 0.58 |
| HB16 | -13.4 | -190.0 | 139.8 | 0.20 | 0.10 | -0.08 | 0.24 | 0.14 | 0.91 |
| HB17 | 115.8 | -261.6 | 46.3 | 0.13 | 0.22 | 0.04 | 0.11 | 0.44 | -0.03 |
| HB18 | 110.5 | 275.4 | 385.2 | -0.27 | -0.12 | -0.14 | -0.20 | -0.26 | -0.08 |
| HB19 | -80.3 | 144.8 | 66.1 | -0.41 | -0.62 | -0.11 | -0.43 | -0.65 | -0.01 |
| HB20 | -170.1 | -446.7 | -431.6 | 0.82 | 0.90* | 0.1 | 0.68* | 0.96* | 0.04 |
| HB21 | 30.4 | 117.1 | 98.8 | -0.38 | -0.60 | -0.08 | -0.31 | -0.63 | -0.11 |
| HB22 | -103.9 | 114.3 | -159.1 | 0.19 | 0.07 | 0 | 0.10 | -0.07 | 0.06 |
| HB23 | 7.6 | -251.4 | -81.7 | 0.28 | 0.37 | 0.15 | 0.27 | 0.43 | 0.01 |
| HB24 | -29.4 | -287.8 | -7.9 | 0.02 | 0.17 | 0.12 | 0.07 | 0.35 | 0.23 |
| HB25 | -75.7 | 131.2 | -96.1 | 0.26 | 0.12 | 0.11 | 0.20 | 0.03 | 0.16 |
| HB26 | 63.7 | -82.8 | -120.7 | -0.22 | 0.05 | 0.13 | -0.25 | 0.06 | 0.08 |
| HB27 | -180.2 | -540.9 | -55.6 | 0.44 | 0.35 | -0.23 | 0.45 | 0.35 | -0.17 |
| HB28 | 110.1 | 310.2 | 126.9 | -0.48 | -0.71 | -0.04 | -0.41 | -0.73 | -0.12 |
| HB29 | -0.9 | -28.4 | 47.9 | -0.10 | 0.27 | -0.09 | -0.07 | 0.27 | -0.05 |
| HB30 | 55.7 | 259.0 | 21.5 | -0.08 | -0.34 | -0.03 | -0.01 | -0.24 | -0.07 |
| HB31 | 17.8 | -287.2 | -116.6 | -0.22 | -0.16 | 0.12 | -0.24 | -0.21 | 0.13 |
| HB32 | -94.0 | -283.8 | -45.2 | 0.36 | 0.20 | -0.05 | 0.30 | 0.11 | -0.05 |
| HB33 | 171.3 | 54.1 | -7.9 | -0.07 | 0.02 | -0.04 | -0.17 | 0.03 | -0.07 |
| HB34 | -80.6 | -41.2 | -157.9 | 0.04 | 0.08 | -0.11 | 0.05 | 0.03 | -0.13 |
| HB35 | 26.1 | 55.9 | 87.8 | 0.03 | -0.02 | -0.11 | 0.16 | 0.00 | -0.03 |
| HB36 | -186.9 | -380.3 | -127.7 | 0.50 | 0.35 | 0.42 | 0.42 | 0.46 | 0.39 |
| HB37 | 179.5 | 505.3 | 76.1 | -0.09 | -0.18 | -0.17 | -0.05 | -0.28 | -0.11 |
| HB38 | 136.2 | 434.5 | -113.3 | -0.02 | 0.06 | -0.11 | -0.13 | 0.11 | -0.02 |
| HB39 | -199.7 | -439.0 | 87.4 | 0.17 | 0.34 | 0.19 | 0.11 | 0.18 | 0.11 |
| HB40 | -37.0 | 168.3 | -20.4 | -0.03 | -0.01 | 0.12 | 0.09 | 0.09 | 0.05 |
| HB41 | -62.8 | 263.7 | 255.7 | -0.32 | -0.32 | -0.21 | -0.29 | -0.47 | -0.15 |
| HB42 | -24.8 | 125.3 | 195.2 | -0.17 | -0.15 | 0.17 | -0.01 | -0.01 | 0.12 |
| HB43 | 127.6 | 200.3 | 87.7 | -0.47 | -0.46 | 0.07 | -0.55 | -0.52 | 0.02 |
| HB44 | 124.0 | 106.9 | 109.8 | -0.26 | -0.32 | -0.37 | -0.25 | -0.42 | -0.3 |
| HB45 | -230.0 | -145.1 | -173.8 | 0.48 | 0.36 | 0.37 | 0.59 | 0.50 | 0.29 |
| HB46 | 68.1 | -30.7 | 90.8 | 0.01 | 0.02 | 0.07 | -0.07 | -0.09 | 0.04 |
| HB47 | 44.1 | 354.9 | 235.6 | 0.10 | 0.08 | -0.32 | 0.13 | 0.22 | -0.24 |
| HB48 | 96.9 | -282.2 | -284.2 | -0.32 | -0.09 | -0.01 | -0.33 | -0.15 | 0.03 |
| HB49 | -5.8 | -269.2 | 32.2 | 0.48 | 0.39 | -0.05 | 0.49 | 0.53 | -0.06 |
| HB50 | -61.2 | -17.8 | -306.3 | 0.25 | 0.39 | 0.07 | 0.21 | 0.37 | 0.01 |
| HB51 | 133.5 | 629.3 | 288.4 | -0.50 | -0.69 | -0.06 | -0.40 | -0.72 | 0 |
| HB52 | 60.5 | -14.3 | -1.5 | -0.36 | -0.17 | 0.1 | -0.41 | -0.24 | 0.14 |
| HB53 | -42.4 | 218.5 | -138.7 | -0.33 | -0.19 | 0.2 | -0.30 | -0.13 | 0.13 |
| HB54 | 107.0 | -40.3 | 99.3 | 0.35 | 0.13 | -0.05 | 0.27 | 0.11 | 0.01 |
| HB55 | -77.0 | -169.7 | -87.9 | 0.05 | 0.13 | -0.03 | 0.02 | 0.15 | -0.04 |
| HB56 | 158.2 | 350.8 | 405.4 | -0.41 | -0.33 | -0.16 | -0.30 | -0.39 | -0.15 |
| HB57 | 95.1 | 53.3 | 115.9 | -0.24 | -0.26 | 0.03 | -0.16 | -0.45 | 0 |
| HB58 | 27.3 | 227.4 | 77.9 | -0.62 | -0.51 | -0.19 | -0.62 | -0.50 | -0.13 |
| HB59 | -131.6 | -586.9* | -137.8 | 0.01 | 0.23 | 0.01 | 0.02 | 0.29 | 0.03 |
| HB60 | -131.3 | -195.6 | -164.8 | 0.66* | 0.35 | -0.02 | 0.58 | 0.33 | -0.01 |
| HB61 | 65.3 | 193.8 | 170.2 | -0.17 | -0.19 | -0.36 | -0.29 | -0.25 | -0.21 |
| HB62 | 48.3 | 173.7 | 362.4 | -0.51 | -0.43 | -0.02 | -0.35 | -0.56 | -0.06 |
| HB63 | 45.5 | -0.6 | -172.9 | 0.51 | 0.39 | 0.18 | 0.45 | 0.34 | 0.1 |
| HB64 | -212.1 | -585.7* | -645.1 | 0.47 | 0.41 | 0.24 | 0.41 | 0.61 | 0.18 |
| HB65 | 191.0 | 432.6 | 382.3 | -0.39 | -0.39 | -0.3 | -0.29 | -0.29 | -0.15 |
| HB66 | -29.3 | -80.6 | -28.3 | 0.25 | 0.18 | 0.01 | 0.19 | 0.19 | -0.08 |
| HB67 | -34.7 | -13.4 | 73.9 | 0.31 | 0.24 | 0.02 | 0.25 | 0.23 | 0.00 |
| HB68 | -49.9 | 481.2 | -71.3 | -0.13 | -0.04 | 0.1 | -0.14 | -0.14 | 0.09 |
| HB69 | 191.1 | 323.4 | 142.9 | -0.38 | -0.56 | -0.1 | -0.32 | -0.81 | -0.06 |
| HB70 | -61.3 | -180.6 | 113.0 | 0.31 | 0.24 | -0.03 | 0.37 | 0.32 | -0.04 |
| HB71 | -29.2 | -6.7 | -151.3 | -0.04 | 0.33 | 0.16 | -0.14 | 0.31 | 0.04 |
| HB72 | 66.4 | 217.2 | 132.7 | -0.22 | -0.28 | -0.16 | -0.25 | -0.23 | -0.05 |
| HB73 | -256.1 | -834.1 | -260.0 | 0.62 | 0.61 | 0.32 | 0.42 | 0.87 | 0.13 |
| HB74 | 15.5 | 189.6 | 170.6 | 0.10 | -0.10 | -0.02 | 0.03 | -0.04 | -0.01 |
| HB75 | 36.8 | 322.3 | 47.2 | -0.39 | -0.14 | -0.11 | -0.26 | -0.24 | -0.06 |
| HB76 | 86.1 | -209.4 | -207.7 | 0.13 | 0.16 | 0.04 | 0.23 | 0.16 | 0.12 |
| HB77 | -232.1 | -506.9 | -226.9 | 0.44 | 0.58 | 0.14 | 0.44 | 0.56 | 0.07 |
| HB78 | 183.7 | 417.9 | -23.7 | -0.54 | -0.22 | -0.02 | -0.50 | -0.31 | 0.12 |
| HB79 | 0.6 | -193.3 | -36.9 | 0.18 | -0.31 | -0.12 | 0.20 | -0.17 | -0.01 |
| HB80 | -102.1 | -366.9 | -62.4 | 0.24 | 0.14 | 0.21 | 0.18 | 0.17 | -0.05 |
| HB81 | -116.4 | -513.1 | -296.1 | 0.66 | 0.36 | 0.27 | 0.59 | 0.40 | 0.2 |
| HB82 | 259.3 | 962.8*** | 69.3 | -0.62 | -0.77 | -0.13 | -0.58 | 0.93* | -0.06 |
| HB83 | -124.7 | -241.0 | -41.7 | -0.05 | 0.11 | -0.12 | -0.01 | 0.05 | -0.08 |
| HB84 | -47.8 | -238.1 | 118.9 | 0.02 | 0.29 | 0.07 | 0.04 | 0.43 | 0.06 |
| HB85 | 98.7 | 533.4 | 714.6 | -0.34 | -0.39 | -0.27 | -0.30 | -0.42 | -0.19 |
| HB86 | 26.1 | -410.9 | 21.2 | 0.17 | 0.38 | 0.08 | 0.18 | 0.42 | 0.11 |
| HB87 | -64.2 | -165.2 | -241.7 | -0.49 | -0.69 | -0.04 | -0.54 | -0.78 | -0.09 |
| HB88 | -10.3 | 385.4 | -79.1 | 0.34 | 0.26 | 0.04 | 0.35 | 0.27 | 0.02 |
| HB89 | -52.4 | -218.4 | -321.0 | 0.30 | 0.26 | 0.09 | 0.29 | 0.22 | 0.1 |
| HB90 | -79.9 | -263.4 | 117.8 | 0.30 | 0.61 | -0.13 | 0.27 | 0.67 | -0.12 |
| HB91 | 61.5 | 197.5 | 340.8 | -0.21 | -0.44 | -0.05 | -0.21 | -0.44 | -0.06 |
| HB92 | 58.4 | 48.2 | -155.3 | -0.19 | -0.13 | 0.05 | -0.13 | -0.05 | 0.03 |
| HB93 | -34.9 | -33.7 | -286.7 | -0.25 | 0.20 | -0.01 | -0.20 | 0.33 | -0.07 |
| HB94 | -80.1 | -23.8 | -145.7 | 0.24 | -0.15 | 0.19 | 0.16 | -0.06 | 0.08 |
| HB95 | 229.9 | 534.2 | 492.7 | -0.25 | -0.03 | -0.12 | -0.14 | -0.09 | -0.04 |
| HB96 | -39.3 | -170.8 | 66.5 | 0.08 | -0.15 | -0.06 | -0.02 | -0.33 | -0.02 |
