# Supplementary material for: Genetic analysis of drought and heat tolerance combined with Striga hermonthica resistance in tropical maize (Zea mays)
Source: PLoS One. 2026 Feb 9;21(2):e0340288. doi: 10.1371/journal.pone.0340288 (PMC12885306; doi:10.1371/journal.pone.0340288)
Supplement: S4 Table — (DOCX) [file pone.0340288.s004.docx]

Supplementary table 4 Mean performance for grain yield of 100 genotypes (96 hybrids and 4 standard checks) tested under Striga free (STUN), Striga infestation (STIN), combined drought and heat stress conditions (CDHS)

| Genotype | GYSTIN (Kgha-1) | GYSTUN (Kgha-1) | GYCDHS (Kgha-1) | Genotype | GYSTIN (Kgha-1) | GYUN (Kgha-1) | GYCDHS (Kgha-1) |
| --- | --- | --- | --- | --- | --- | --- | --- |
| HB1 | 3484.4 | 3972.0 | 1366.7 | HB51 | 4481.5 | 4479.1 | 2304.4 |
| HB2 | 3909.0 | 4453.1 | 1348.0 | HB52 | 2782.8 | 3471.1 | 1919.3 |
| HB3 | 3939.3 | 4312.3 | 1109.6 | HB53 | 3699.1 | 4632.6 | 2455.5 |
| HB4 | 3190.5 | 4067.1 | 4023.6 | HB54 | 3752.3 | 4773.3 | 3294.6 |
| HB5 | 3310.1 | 3452.0 | 1093.7 | HB55 | 3380.4 | 3983.3 | 2466.5 |
| HB6 | 3417.9 | 4099.5 | 467.5 | HB56 | 4088.4 | 4322.8 | 3556.9 |
| HB7 | 3036.3 | 3592.1 | 1828.3 | HB57 | 3500.5 | 3690.1 | 4025.4 |
| HB8 | 3229.3 | 3228.0 | 552.9 | HB58 | 3671.1 | 3756.9 | 2122.6 |
| HB9 | 3166.4 | 3343.6 | 4068.3 | HB59 | 2628.6 | 2743.5 | 631.5 |
| HB10 | 3423.1 | 3632.0 | 3313.4 | HB60 | 2325.3 | 2204.1 | 1362.9 |
| HB11 | 3634.8 | 3433.1 | 3382.5 | HB61 | 3511.4 | 4187.4 | 3556.2 |
| HB12 | 3181.5 | 3662.8 | 2893.6 | HB62 | 3892.9 | 4160.3 | 2864.6 |
| HB13 | 2387.1 | 2568.6 | 1315.5 | HB63 | 3036.0 | 3250.9 | 1503.7 |
| HB14 | 2317.9 | 2679.1 | 1400.4 | HB64 | 1893.9 | 2053.8 | 1033.3 |
| HB15 | 2625.6 | 2644.0 | 684.2 | HB65 | 4194.9 | 4613.9 | 4183.0 |
| HB16 | 2623.3 | 3414.1 | 1403.5 | HB66 | 3732.8 | 4382.8 | 2553.1 |
| HB17 | 2891.9 | 4455.9 | 2513.2 | HB67 | 3788.0 | 4280.0 | 4474.8 |
| HB18 | 3845.9 | 4577.5 | 3816.6 | HB68 | 4376.6 | 4077.6 | 3574.5 |
| HB19 | 2816.9 | 2420.9 | 1041.2 | HB69 | 3601.0 | 4495.5 | 3211.2 |
| HB20 | 2324.5 | 2241.6 | 1516.0 | HB70 | 3531.0 | 4719.3 | 3949.2 |
| HB21 | 3203.5 | 3491.5 | 1183.6 | HB71 | 3610.9 | 4391.5 | 1396.8 |
| HB22 | 3699.4 | 3759.9 | 3060.5 | HB72 | 3801.8 | 4330.0 | 3142.5 |
| HB23 | 2214.3 | 2456.4 | 2146.0 | HB73 | 1470.3 | 1893.9 | 274.3 |
| HB24 | 2287.1 | 2507.3 | 245.1 | HB74 | 3716.8 | 3903.6 | 3868.6 |
| HB25 | 3709.0 | 3875.1 | 958.9 | HB75 | 4101.9 | 3682.3 | 2081.3 |
| HB26 | 3618.4 | 4411.3 | 1539.7 | HB76 | 2708.9 | 3242.0 | 896.5 |
| HB27 | 1747.4 | 1770.4 | 1778.1 | HB77 | 2156.9 | 2267.5 | 517.5 |
| HB28 | 3001.4 | 2818.5 | 647.4 | HB78 | 3871.0 | 4283.3 | 2429.5 |
| HB29 | 3365.9 | 3877.4 | 2156.1 | HB79 | 3167.0 | 3142.1 | 2145.8 |
| HB30 | 3930.3 | 4345.5 | 2637.2 | HB80 | 2783.3 | 3128.0 | 1028.7 |
| HB31 | 2154.1 | 2688.5 | 2046.7 | HB81 | 2490.8 | 2769.0 | 1609.0 |
| HB32 | 2378.3 | 2769.0 | 1219.8 | HB82 | 5214.0 | 5368.6 | 2119.5 |
| HB33 | 3760.9 | 4134.5 | 2748.3 | HB83 | 3215.6 | 3826.8 | 4129.5 |
| HB34 | 3602.4 | 3724.9 | 1542.9 | HB84 | 2853.8 | 3025.8 | 1203.5 |
| HB35 | 3423.1 | 3490.0 | 2741.0 | HB85 | 3963.4 | 3022.3 | 6090.1 |
| HB36 | 2114.3 | 2482.8 | 361.1 | HB86 | 3345.0 | 4828.5 | 3245.2 |
| HB37 | 5012.6 | 5031.0 | 2933.9 | HB87 | 2769.1 | 4193.4 | 3589.4 |
| HB38 | 4678.9 | 5013.1 | 410.1 | HB88 | 4258.6 | 3529.1 | 2048.1 |
| HB39 | 2943.6 | 3546.6 | 4113.8 | HB89 | 2622.3 | 2959.9 | 2090.4 |
| HB40 | 3706.1 | 3970.1 | 540.4 | HB90 | 2904.5 | 3531.6 | 1576.8 |
| HB41 | 4202.1 | 4471.1 | 5705.8 | HB91 | 3879.4 | 3889.0 | 5142.7 |
| HB42 | 4239.4 | 4953.5 | 3869.5 | HB92 | 3047.4 | 3615.4 | 2340.0 |
| HB43 | 3808.1 | 4562.5 | 5609.3 | HB93 | 3181.1 | 3324.0 | 418.6 |
| HB44 | 3395.3 | 4769.0 | 2696.8 | HB94 | 3656.6 | 4429.3 | 1955.8 |
| HB45 | 3497.8 | 2942.4 | 304.8 | HB95 | 4783.8 | 5398.4 | 6715.8 |
| HB46 | 3654.4 | 4532.8 | 2964.7 | HB96 | 3244.8 | 4014.5 | 1731.0 |
| HB47 | 3483.1 | 3392.3 | 5384.1 | HB97 | 3655.8 | 3210.1 | 3336.7 |
| HB48 | 2557.5 | 3643.5 | 614.4 | HB98 | 3022.8 | 1966.1 | 2634.4 |
| HB49 | 3268.5 | 4657.3 | 2910.4 | HB99 | 7102.8 | 7066.1 | 5640.3 |
| HB50 | 3780.4 | 4023.6 | 2167.1 | HB100 | 5920.8 | 5927.1 | 2404.0 |
|  |  |  |  | Mean | 3395.8 | 3748.0 | 2398.0 |
|  |  |  |  | LSD (%) | 937.9 | 918.2 | 2225.3 |
