# Supplementary material for: Genetic analysis of drought and heat tolerance combined with Striga hermonthica resistance in tropical maize (Zea mays)
Source: PLoS One. 2026 Feb 9;21(2):e0340288. doi: 10.1371/journal.pone.0340288 (PMC12885306; doi:10.1371/journal.pone.0340288)
Supplement: S5 Table — (DOCX) [file pone.0340288.s005.docx]

Supplementary Table 5 Stress tolerant indices under Striga infested environment

| Hybrid | GYSTIN | GYSTNO | GMP | MP | HM | STI | YI | YSI |
| --- | --- | --- | --- | --- | --- | --- | --- | --- |
| SC1 | 3484.4 | 3972.0 | 3720.2 | 3728.2 | 3712.3 | 3692.6 | 1.03 | 0.88 |
| SC2 | 3909.0 | 4453.1 | 4172.2 | 4181.1 | 4163.3 | 4644.4 | 1.15 | 0.88 |
| SC3 | 3939.3 | 4312.3 | 4121.6 | 4125.8 | 4117.4 | 4532.4 | 1.16 | 0.91 |
| SC4 | 3190.5 | 4067.1 | 3602.2 | 3628.8 | 3575.9 | 3462.1 | 0.94 | 0.78 |
| SC5 | 3310.1 | 3452.0 | 3380.3 | 3381.1 | 3379.6 | 3048.7 | 0.97 | 0.96 |
| SC6 | 3417.9 | 4099.5 | 3743.2 | 3758.7 | 3727.8 | 3738.4 | 1.01 | 0.83 |
| SC7 | 3036.3 | 3592.1 | 3302.5 | 3314.2 | 3290.9 | 2910.0 | 0.89 | 0.85 |
| SC8 | 3229.3 | 3228.0 | 3228.6 | 3228.7 | 3228.6 | 2781.3 | 0.95 | 1.00 |
| SC9 | 3166.4 | 3343.6 | 3253.8 | 3255.0 | 3252.6 | 2824.8 | 0.93 | 0.95 |
| SC10 | 3423.1 | 3632.0 | 3526.0 | 3527.6 | 3524.5 | 3317.2 | 1.01 | 0.94 |
| SC11 | 3634.8 | 3433.1 | 3532.5 | 3534.0 | 3531.1 | 3329.4 | 1.07 | 1.06 |
| SC12 | 3181.5 | 3662.8 | 3413.7 | 3422.2 | 3405.2 | 3109.2 | 0.94 | 0.87 |
| SC13 | 2387.1 | 2568.6 | 2476.2 | 2477.9 | 2474.5 | 1635.9 | 0.70 | 0.93 |
| SC14 | 2317.9 | 2679.1 | 2492.0 | 2498.5 | 2485.4 | 1656.9 | 0.68 | 0.87 |
| SC15 | 2625.6 | 2644.0 | 2634.8 | 2634.8 | 2634.8 | 1852.2 | 0.77 | 0.99 |
| SC16 | 2623.3 | 3414.1 | 2992.7 | 3018.7 | 2966.9 | 2389.6 | 0.77 | 0.77 |
| SC17 | 2891.9 | 4455.9 | 3589.7 | 3673.9 | 3507.4 | 3438.1 | 0.85 | 0.65 |
| SC18 | 3845.9 | 4577.5 | 4195.8 | 4211.7 | 4179.9 | 4697.1 | 1.13 | 0.84 |
| SC19 | 2816.9 | 2420.9 | 2611.4 | 2618.9 | 2603.9 | 1819.5 | 0.83 | 1.16 |
| SC20 | 2324.5 | 2241.6 | 2282.7 | 2283.1 | 2282.3 | 1390.2 | 0.68 | 1.04 |
| SC21 | 3203.5 | 3491.5 | 3344.4 | 3347.5 | 3341.3 | 2984.3 | 0.94 | 0.92 |
| SC22 | 3699.4 | 3759.9 | 3729.5 | 3729.7 | 3729.4 | 3711.1 | 1.09 | 0.98 |
| SC23 | 2214.3 | 2456.4 | 2332.2 | 2335.4 | 2329.1 | 1451.2 | 0.65 | 0.90 |
| SC24 | 2287.1 | 2507.3 | 2394.7 | 2397.2 | 2392.1 | 1530.0 | 0.67 | 0.91 |
| SC25 | 3709.0 | 3875.1 | 3791.1 | 3792.1 | 3790.2 | 3834.8 | 1.09 | 0.96 |
| SC26 | 3618.4 | 4411.3 | 3995.2 | 4014.9 | 3975.7 | 4258.8 | 1.07 | 0.82 |
| SC27 | 1747.4 | 1770.4 | 1758.9 | 1758.9 | 1758.8 | 825.4 | 0.51 | 0.99 |
| SC28 | 3001.4 | 2818.5 | 2908.5 | 2910.0 | 2907.1 | 2257.1 | 0.88 | 1.06 |
| SC29 | 3365.9 | 3877.4 | 3612.6 | 3621.7 | 3603.6 | 3482.1 | 0.99 | 0.87 |
| SC30 | 3930.3 | 4345.5 | 4132.7 | 4137.9 | 4127.5 | 4556.9 | 1.16 | 0.90 |
| SC31 | 2154.1 | 2688.5 | 2406.5 | 2421.3 | 2391.8 | 1545.2 | 0.63 | 0.80 |
| SC32 | 2378.3 | 2769.0 | 2566.2 | 2573.7 | 2558.8 | 1757.1 | 0.70 | 0.86 |
| SC33 | 3760.9 | 4134.5 | 3943.3 | 3947.7 | 3938.9 | 4148.7 | 1.11 | 0.91 |
| SC34 | 3602.4 | 3724.9 | 3663.1 | 3663.7 | 3662.6 | 3580.2 | 1.06 | 0.97 |
| SC35 | 3423.1 | 3490.0 | 3456.4 | 3456.6 | 3456.2 | 3187.5 | 1.01 | 0.98 |
| SC36 | 2114.3 | 2482.8 | 2291.2 | 2298.6 | 2283.8 | 1400.6 | 0.62 | 0.85 |
| SC37 | 5012.6 | 5031.0 | 5021.8 | 5021.8 | 5021.8 | 6728.5 | 1.48 | 1.00 |
| SC38 | 4678.9 | 5013.1 | 4843.1 | 4846.0 | 4840.2 | 6258.2 | 1.38 | 0.93 |
| SC39 | 2943.6 | 3546.6 | 3231.1 | 3245.1 | 3217.1 | 2785.4 | 0.87 | 0.83 |
| SC40 | 3706.1 | 3970.1 | 3835.8 | 3838.1 | 3833.6 | 3925.7 | 1.09 | 0.93 |
| SC41 | 4202.1 | 4471.1 | 4334.5 | 4336.6 | 4332.4 | 5012.8 | 1.24 | 0.94 |
| SC42 | 4239.4 | 4953.5 | 4582.6 | 4596.5 | 4568.7 | 5603.0 | 1.25 | 0.86 |
| SC43 | 3808.1 | 4562.5 | 4168.3 | 4185.3 | 4151.3 | 4635.7 | 1.12 | 0.83 |
| SC44 | 3395.3 | 4769.0 | 4024.0 | 4082.2 | 3966.6 | 4320.2 | 1.00 | 0.71 |
| SC45 | 3497.8 | 2942.4 | 3208.1 | 3220.1 | 3196.2 | 2746.0 | 1.03 | 1.19 |
| SC46 | 3654.4 | 4532.8 | 4070.0 | 4093.6 | 4046.5 | 4419.6 | 1.08 | 0.81 |
| SC47 | 3483.1 | 3392.3 | 3437.4 | 3437.7 | 3437.1 | 3152.5 | 1.03 | 1.03 |
| SC48 | 2557.5 | 3643.5 | 3052.6 | 3100.5 | 3005.4 | 2486.2 | 0.75 | 0.70 |
| SC49 | 3268.5 | 4657.3 | 3901.6 | 3962.9 | 3841.2 | 4061.5 | 0.96 | 0.70 |
| SC50 | 3780.4 | 4023.6 | 3900.1 | 3902.0 | 3898.2 | 4058.4 | 1.11 | 0.94 |
| SC51 | 4481.5 | 4479.1 | 4480.3 | 4480.3 | 4480.3 | 5355.7 | 1.32 | 1.00 |
| SC52 | 2782.8 | 3471.1 | 3108.0 | 3127.0 | 3089.1 | 2577.2 | 0.82 | 0.80 |
| SC53 | 3699.1 | 4632.6 | 4139.6 | 4165.9 | 4113.6 | 4572.2 | 1.09 | 0.80 |
| SC54 | 3752.3 | 4773.3 | 4232.1 | 4262.8 | 4201.7 | 4778.8 | 1.10 | 0.79 |
| SC55 | 3380.4 | 3983.3 | 3669.5 | 3681.9 | 3657.2 | 3592.6 | 1.00 | 0.85 |
| SC56 | 4088.4 | 4322.8 | 4204.0 | 4205.6 | 4202.3 | 4715.4 | 1.20 | 0.95 |
| SC57 | 3500.5 | 3690.1 | 3594.0 | 3595.3 | 3592.8 | 3446.4 | 1.03 | 0.95 |
| SC58 | 3671.1 | 3756.9 | 3713.8 | 3714.0 | 3713.5 | 3679.8 | 1.08 | 0.98 |
| SC59 | 2628.6 | 2743.5 | 2685.4 | 2686.1 | 2684.8 | 1924.1 | 0.77 | 0.96 |
| SC60 | 2325.3 | 2204.1 | 2263.9 | 2264.7 | 2263.1 | 1367.4 | 0.68 | 1.05 |
| SC61 | 3511.4 | 4187.4 | 3834.5 | 3849.4 | 3819.7 | 3923.1 | 1.03 | 0.84 |
| SC62 | 3892.9 | 4160.3 | 4024.4 | 4026.6 | 4022.2 | 4321.1 | 1.15 | 0.94 |
| SC63 | 3036.0 | 3250.9 | 3141.6 | 3143.5 | 3139.8 | 2633.3 | 0.89 | 0.93 |
| SC64 | 1893.9 | 2053.8 | 1972.2 | 1973.9 | 1970.6 | 1037.8 | 0.56 | 0.92 |
| SC65 | 4194.9 | 4613.9 | 4399.4 | 4404.4 | 4394.4 | 5164.0 | 1.24 | 0.91 |
| SC66 | 3732.8 | 4382.8 | 4044.8 | 4057.8 | 4031.8 | 4365.0 | 1.10 | 0.85 |
| SC67 | 3788.0 | 4280.0 | 4026.5 | 4034.0 | 4019.0 | 4325.7 | 1.12 | 0.89 |
| SC68 | 4376.6 | 4077.6 | 4224.5 | 4227.1 | 4221.8 | 4761.5 | 1.29 | 1.07 |
| SC69 | 3601.0 | 4495.5 | 4023.5 | 4048.3 | 3998.8 | 4319.2 | 1.06 | 0.80 |
| SC70 | 3531.0 | 4719.3 | 4082.1 | 4125.2 | 4039.6 | 4446.1 | 1.04 | 0.75 |
| SC71 | 3610.9 | 4391.5 | 3982.1 | 4001.2 | 3963.1 | 4230.9 | 1.06 | 0.82 |
| SC72 | 3801.8 | 4330.0 | 4057.3 | 4065.9 | 4048.7 | 4392.2 | 1.12 | 0.88 |
| SC73 | 1470.3 | 1893.9 | 1668.7 | 1682.1 | 1655.4 | 743.0 | 0.43 | 0.78 |
| SC74 | 3716.8 | 3903.6 | 3809.1 | 3810.2 | 3807.9 | 3871.1 | 1.09 | 0.95 |
| SC75 | 4101.9 | 3682.3 | 3886.4 | 3892.1 | 3880.8 | 4030.0 | 1.21 | 1.11 |
| SC76 | 2708.9 | 3242.0 | 2963.5 | 2975.5 | 2951.6 | 2343.2 | 0.80 | 0.84 |
| SC77 | 2156.9 | 2267.5 | 2211.5 | 2212.2 | 2210.8 | 1304.9 | 0.64 | 0.95 |
| SC78 | 3871.0 | 4283.3 | 4071.9 | 4077.2 | 4066.7 | 4423.9 | 1.14 | 0.90 |
| SC79 | 3167.0 | 3142.1 | 3154.5 | 3154.6 | 3154.5 | 2655.0 | 0.93 | 1.01 |
| SC80 | 2783.3 | 3128.0 | 2950.6 | 2955.7 | 2945.6 | 2322.9 | 0.82 | 0.89 |
| SC81 | 2490.8 | 2769.0 | 2626.2 | 2629.9 | 2622.5 | 1840.2 | 0.73 | 0.90 |
| SC82 | 5214.0 | 5368.6 | 5290.7 | 5291.3 | 5290.2 | 7468.5 | 1.54 | 0.97 |
| SC83 | 3215.6 | 3826.8 | 3507.9 | 3521.2 | 3494.7 | 3283.2 | 0.95 | 0.84 |
| SC84 | 2853.8 | 3025.8 | 2938.5 | 2939.8 | 2937.3 | 2303.9 | 0.84 | 0.94 |
| SC85 | 3963.4 | 3022.3 | 3461.0 | 3492.9 | 3429.5 | 3196.0 | 1.17 | 1.31 |
| SC86 | 3345.0 | 4828.5 | 4018.9 | 4086.8 | 3952.1 | 4309.3 | 0.99 | 0.69 |
| SC87 | 2769.1 | 4193.4 | 3407.6 | 3481.3 | 3335.6 | 3098.2 | 0.82 | 0.66 |
| SC88 | 4258.6 | 3529.1 | 3876.7 | 3893.9 | 3859.7 | 4009.9 | 1.25 | 1.21 |
| SC89 | 2622.3 | 2959.9 | 2786.0 | 2791.1 | 2780.9 | 2070.9 | 0.77 | 0.89 |
| SC90 | 2904.5 | 3531.6 | 3202.7 | 3218.1 | 3187.5 | 2736.8 | 0.86 | 0.82 |
| SC91 | 3879.4 | 3889.0 | 3884.2 | 3884.2 | 3884.2 | 4025.3 | 1.14 | 1.00 |
| SC92 | 3047.4 | 3615.4 | 3319.3 | 3331.4 | 3307.2 | 2939.6 | 0.90 | 0.84 |
| SC93 | 3181.1 | 3324.0 | 3251.8 | 3252.6 | 3251.0 | 2821.2 | 0.94 | 0.96 |
| SC94 | 3656.6 | 4429.3 | 4024.4 | 4043.0 | 4006.0 | 4321.3 | 1.08 | 0.83 |
| SC95 | 4783.8 | 5398.4 | 5081.8 | 5091.1 | 5072.6 | 6890.3 | 1.41 | 0.89 |
| SC96 | 3244.8 | 4014.5 | 3609.2 | 3629.7 | 3588.8 | 3475.5 | 0.96 | 0.81 |
| CH97 | 3655.8 | 3210.1 | 3425.7 | 3433.0 | 3418.5 | 3131.1 | 1.08 | 1.14 |
| CH98 | 3022.8 | 1966.1 | 2437.9 | 2494.5 | 2382.5 | 1585.7 | 0.89 | 1.54 |
| COH99 | 7102.8 | 7066.1 | 7084.4 | 7084.5 | 7084.4 | 13390.9 | 2.09 | 1.01 |
| COH100 | 5920.8 | 5927.1 | 5923.9 | 5924.0 | 5923.9 | 9363.2 | 1.74 | 1.00 |
| Mean | 3395.8 | 3748.0 |  |  |  |  |  |  |
