# Supplementary material for: Genetic analysis of drought and heat tolerance combined with Striga hermonthica resistance in tropical maize (Zea mays)
Source: PLoS One. 2026 Feb 9;21(2):e0340288. doi: 10.1371/journal.pone.0340288 (PMC12885306; doi:10.1371/journal.pone.0340288)
Supplement: S6 Table — (DOCX) [file pone.0340288.s006.docx]

Supplementary table 6 Stress tolerant indices under combined heat and drought environment

| Hybrid | GYDHS | GYFIR | GMP | MP | HM | STI | YI | YSI |
| --- | --- | --- | --- | --- | --- | --- | --- | --- |
| SC1 | 1366.7 | 4953.3 | 2602 | 3160 | 2142 | 0.42 | 0.57 | 0.28 |
| SC2 | 1348.0 | 4195.2 | 2378 | 2772 | 2040 | 0.35 | 0.56 | 0.32 |
| SC3 | 1109.6 | 4571.9 | 2252 | 2841 | 1786 | 0.32 | 0.46 | 0.24 |
| SC4 | 4023.6 | 4677.2 | 4338 | 4350 | 4326 | 1.18 | 1.68 | 0.86 |
| SC5 | 1093.7 | 5063.0 | 2353 | 3078 | 1799 | 0.35 | 0.46 | 0.22 |
| SC6 | 467.5 | 3910.0 | 1352 | 2189 | 835 | 0.11 | 0.19 | 0.12 |
| SC7 | 1828.3 | 3812.1 | 2640 | 2820 | 2471 | 0.44 | 0.76 | 0.48 |
| SC8 | 552.9 | 5172.2 | 1691 | 2863 | 999 | 0.18 | 0.23 | 0.11 |
| SC9 | 4068.3 | 3933.4 | 4000 | 4001 | 4000 | 1.00 | 1.70 | 1.03 |
| SC10 | 3313.4 | 3762.3 | 3531 | 3538 | 3524 | 0.78 | 1.38 | 0.88 |
| SC11 | 3382.5 | 3293.3 | 3338 | 3338 | 3337 | 0.70 | 1.41 | 1.03 |
| SC12 | 2893.6 | 2859.7 | 2877 | 2877 | 2877 | 0.52 | 1.21 | 1.01 |
| SC13 | 1315.5 | 3769.6 | 2227 | 2543 | 1950 | 0.31 | 0.55 | 0.35 |
| SC14 | 1400.4 | 2451.8 | 1853 | 1926 | 1783 | 0.22 | 0.58 | 0.57 |
| SC15 | 684.2 | 3381.6 | 1521 | 2033 | 1138 | 0.15 | 0.29 | 0.20 |
| SC16 | 1403.5 | 3119.4 | 2092 | 2261 | 1936 | 0.27 | 0.59 | 0.45 |
| SC17 | 2513.2 | 5266.0 | 3638 | 3890 | 3402 | 0.83 | 1.05 | 0.48 |
| SC18 | 3816.6 | 4862.5 | 4308 | 4340 | 4277 | 1.16 | 1.59 | 0.78 |
| SC19 | 1041.2 | 2395.8 | 1579 | 1718 | 1452 | 0.16 | 0.43 | 0.43 |
| SC20 | 1516.0 | 1700.6 | 1606 | 1608 | 1603 | 0.16 | 0.63 | 0.89 |
| SC21 | 1183.6 | 3441.4 | 2018 | 2312 | 1761 | 0.26 | 0.49 | 0.34 |
| SC22 | 3060.5 | 3786.3 | 3404 | 3423 | 3385 | 0.73 | 1.28 | 0.81 |
| SC23 | 2146.0 | 2783.6 | 2444 | 2465 | 2424 | 0.37 | 0.89 | 0.77 |
| SC24 | 245.1 | 2395.7 | 766 | 1320 | 445 | 0.04 | 0.10 | 0.10 |
| SC25 | 958.9 | 4460.4 | 2068 | 2710 | 1578 | 0.27 | 0.40 | 0.21 |
| SC26 | 1539.7 | 3822.1 | 2426 | 2681 | 2195 | 0.37 | 0.64 | 0.40 |
| SC27 | 1778.1 | 2857.3 | 2254 | 2318 | 2192 | 0.32 | 0.74 | 0.62 |
| SC28 | 647.4 | 3643.3 | 1536 | 2145 | 1099 | 0.15 | 0.27 | 0.18 |
| SC29 | 2156.1 | 5913.7 | 3571 | 4035 | 3160 | 0.80 | 0.90 | 0.36 |
| SC30 | 2637.2 | 5026.9 | 3641 | 3832 | 3459 | 0.83 | 1.10 | 0.52 |
| SC31 | 2046.7 | 2824.9 | 2405 | 2436 | 2374 | 0.36 | 0.85 | 0.72 |
| SC32 | 1219.8 | 3155.4 | 1962 | 2188 | 1759 | 0.24 | 0.51 | 0.39 |
| SC33 | 2748.3 | 5091.6 | 3741 | 3920 | 3570 | 0.88 | 1.15 | 0.54 |
| SC34 | 1542.9 | 3925.3 | 2461 | 2734 | 2215 | 0.38 | 0.64 | 0.39 |
| SC35 | 2741.0 | 4383.5 | 3466 | 3562 | 3373 | 0.75 | 1.14 | 0.63 |
| SC36 | 361.1 | 3065.5 | 1052 | 1713 | 646 | 0.07 | 0.15 | 0.12 |
| SC37 | 2933.9 | 4058.0 | 3450 | 3496 | 3406 | 0.75 | 1.22 | 0.72 |
| SC38 | 410.1 | 4421.3 | 1347 | 2416 | 751 | 0.11 | 0.17 | 0.09 |
| SC39 | 4113.8 | 3195.0 | 3625 | 3654 | 3597 | 0.83 | 1.72 | 1.29 |
| SC40 | 540.4 | 4565.4 | 1571 | 2553 | 966 | 0.15 | 0.23 | 0.12 |
| SC41 | 5705.8 | 5311.5 | 5505 | 5509 | 5502 | 1.90 | 2.38 | 1.07 |
| SC42 | 3869.5 | 4631.4 | 4233 | 4250 | 4216 | 1.12 | 1.61 | 0.84 |
| SC43 | 5609.3 | 4515.5 | 5033 | 5062 | 5003 | 1.59 | 2.34 | 1.24 |
| SC44 | 2696.8 | 3951.6 | 3264 | 3324 | 3206 | 0.67 | 1.12 | 0.68 |
| SC45 | 304.8 | 4864.0 | 1218 | 2584 | 574 | 0.09 | 0.13 | 0.06 |
| SC46 | 2964.7 | 4395.7 | 3610 | 3680 | 3541 | 0.82 | 1.24 | 0.67 |
| SC47 | 5384.1 | 4493.2 | 4919 | 4939 | 4898 | 1.52 | 2.25 | 1.20 |
| SC48 | 614.4 | 3616.1 | 1491 | 2115 | 1050 | 0.14 | 0.26 | 0.17 |
| SC49 | 2910.4 | 3502.0 | 3193 | 3206 | 3179 | 0.64 | 1.21 | 0.83 |
| SC50 | 2167.1 | 5830.0 | 3554 | 3999 | 3160 | 0.79 | 0.90 | 0.37 |
| SC51 | 2304.4 | 5296.3 | 3494 | 3800 | 3211 | 0.79 | 0.96 | 0.44 |
| SC52 | 1919.3 | 4215.8 | 2845 | 3068 | 2638 | 0.77 | 0.80 | 0.46 |
| SC53 | 2455.5 | 3919.9 | 3102 | 3188 | 3020 | 0.51 | 1.02 | 0.63 |
| SC54 | 3294.6 | 3894.1 | 3582 | 3594 | 3569 | 0.60 | 1.37 | 0.85 |
| SC55 | 2466.5 | 5347.1 | 3632 | 3907 | 3376 | 0.81 | 1.03 | 0.46 |
| SC56 | 3556.9 | 4980.2 | 4209 | 4269 | 4150 | 0.83 | 1.48 | 0.71 |
| SC57 | 4025.4 | 3753.3 | 3887 | 3889 | 3885 | 1.11 | 1.68 | 1.07 |
| SC58 | 2122.6 | 5221.2 | 3329 | 3672 | 3018 | 0.95 | 0.89 | 0.41 |
| SC59 | 631.5 | 3470.7 | 1480 | 2051 | 1069 | 0.70 | 0.26 | 0.18 |
| SC60 | 1362.9 | 2450.4 | 1827 | 1907 | 1752 | 0.14 | 0.57 | 0.56 |
| SC61 | 3556.2 | 4743.1 | 4107 | 4150 | 4065 | 0.21 | 1.48 | 0.75 |
| SC62 | 2864.6 | 4811.8 | 3713 | 3838 | 3591 | 1.06 | 1.19 | 0.60 |
| SC63 | 1503.7 | 1821.0 | 1655 | 1662 | 1647 | 0.87 | 0.63 | 0.83 |
| SC64 | 1033.3 | 2469.2 | 1597 | 1751 | 1457 | 0.17 | 0.43 | 0.42 |
| SC65 | 4183.0 | 4031.4 | 4107 | 4107 | 4106 | 0.16 | 1.74 | 1.04 |
| SC66 | 2553.1 | 2156.3 | 2346 | 2355 | 2338 | 1.06 | 1.06 | 1.18 |
| SC67 | 4474.8 | 3599.4 | 4013 | 4037 | 3990 | 0.35 | 1.87 | 1.24 |
| SC68 | 3574.5 | 4174.1 | 3863 | 3874 | 3851 | 1.01 | 1.49 | 0.86 |
| SC69 | 3211.2 | 4816.4 | 3933 | 4014 | 3853 | 0.94 | 1.34 | 0.67 |
| SC70 | 3949.2 | 3760.1 | 3853 | 3855 | 3852 | 0.97 | 1.65 | 1.05 |
| SC71 | 1396.8 | 5409.2 | 2749 | 3403 | 2220 | 0.93 | 0.58 | 0.26 |
| SC72 | 3142.5 | 5073.6 | 3993 | 4108 | 3881 | 0.47 | 1.31 | 0.62 |
| SC73 | 274.3 | 2073.2 | 754 | 1174 | 485 | 1.00 | 0.11 | 0.13 |
| SC74 | 3868.6 | 3164.1 | 3499 | 3516 | 3481 | 0.04 | 1.61 | 1.22 |
| SC75 | 2081.3 | 4094.9 | 2919 | 3088 | 2760 | 0.77 | 0.87 | 0.51 |
| SC76 | 896.5 | 3405.2 | 1747 | 2151 | 1419 | 0.19 | 0.37 | 0.26 |
| SC77 | 517.5 | 2560.6 | 1151 | 1539 | 861 | 0.08 | 0.22 | 0.20 |
| SC78 | 2429.5 | 4034.4 | 3131 | 3232 | 3033 | 0.62 | 1.01 | 0.60 |
| SC79 | 2145.8 | 3842.8 | 2872 | 2994 | 2754 | 0.52 | 0.89 | 0.56 |
| SC80 | 1028.7 | 3359.8 | 1859 | 2194 | 1575 | 0.22 | 0.43 | 0.31 |
| SC81 | 1609.0 | 3293.0 | 2302 | 2451 | 2162 | 0.33 | 0.67 | 0.49 |
| SC82 | 2119.5 | 4210.0 | 2987 | 3165 | 2820 | 0.56 | 0.88 | 0.50 |
| SC83 | 4129.5 | 3072.5 | 3562 | 3601 | 3523 | 0.80 | 1.72 | 1.34 |
| SC84 | 1203.5 | 3046.0 | 1915 | 2125 | 1725 | 0.23 | 0.50 | 0.40 |
| SC85 | 6090.1 | 4178.0 | 5044 | 5134 | 4956 | 1.60 | 2.54 | 1.46 |
| SC86 | 3245.2 | 3037.5 | 3140 | 3141 | 3138 | 0.62 | 1.35 | 1.07 |
| SC87 | 3589.4 | 3637.5 | 3613 | 3613 | 3613 | 0.82 | 1.50 | 0.99 |
| SC88 | 2048.1 | 3519.0 | 2685 | 2784 | 2589 | 0.45 | 0.85 | 0.58 |
| SC89 | 2090.4 | 3942.0 | 2871 | 3016 | 2732 | 0.52 | 0.87 | 0.53 |
| SC90 | 1576.8 | 4652.5 | 2709 | 3115 | 2355 | 0.46 | 0.66 | 0.34 |
| SC91 | 5142.7 | 4628.5 | 4879 | 4886 | 4872 | 1.49 | 2.14 | 1.11 |
| SC92 | 2340.0 | 3757.8 | 2965 | 3049 | 2884 | 0.55 | 0.98 | 0.62 |
| SC93 | 418.6 | 4079.3 | 1307 | 2249 | 759 | 0.11 | 0.17 | 0.10 |
| SC94 | 1955.8 | 4671.5 | 3023 | 3314 | 2757 | 0.57 | 0.82 | 0.42 |
| SC95 | 6715.8 | 5446.0 | 6048 | 6081 | 6015 | 2.30 | 2.80 | 1.23 |
| SC96 | 1731.0 | 3575.2 | 2488 | 2653 | 2333 | 0.39 | 0.72 | 0.48 |
| CH97 | 3336.7 | 6120.1 | 4519 | 4728 | 4319 | 1.28 | 1.39 | 0.55 |
| CH98 | 2634.4 | 5639.8 | 3855 | 4137 | 3591 | 0.93 | 1.10 | 0.47 |
| COH99 | 5640.3 | 4982.1 | 5301 | 5311 | 5291 | 1.76 | 2.35 | 1.13 |
| COH100 | 2404.0 | 4619.4 | 3332 | 3512 | 3162 | 0.70 | 1.00 | 0.52 |
| Mean | 2398.0 | 3991.4 |  |  |  |  |  |  |
